# Aging gut microbiota of wild macaques are equally diverse, less stable, but progressively personalized

**DOI:** 10.1186/s40168-022-01283-2

**Published:** 2022-06-19

**Authors:** Baptiste Sadoughi, Dominik Schneider, Rolf Daniel, Oliver Schülke, Julia Ostner

**Affiliations:** 1 Department of Behavioral Ecology, Johann-Friedrich-Blumenbach Institute for Zoology and Anthropology, Georg-August-University Göttingen, Kellnerweg 6, D-37077 Göttingen, Germany; 2 Research Group Primate Social Evolution, German Primate Center, Leibniz Institute for Primate Research, Göttingen, Germany; 3 Genomic and Applied Microbiology and Göttingen Genomics Laboratory, Institute of Microbiology and Genetics, Georg-August-University Göttingen, Göttingen, Germany; 4 Leibniz ScienceCampus Primate Cognition, German Primate Center, Leibniz Institute for Primate Research, Göttingen, Germany

**Keywords:** Aging, Senescence, Stability, Gut bacteria, Commensals, Social transmission, Personalized, Primate, Microbiome, Dysbiosis

## Abstract

**Background:**

Pronounced heterogeneity of age trajectories has been identified as a hallmark of the gut microbiota in humans and has been explained by marked changes in lifestyle and health condition. Comparatively, age-related personalization of microbiota is understudied in natural systems limiting our comprehension of patterns observed in humans from ecological and evolutionary perspectives.

**Results:**

Here, we tested age-related changes in the diversity, stability, and composition of the gut bacterial community using 16S rRNA gene sequencing with dense repeated sampling over three seasons in a cross-sectional age sample of adult female Assamese macaques (*Macaca assamensis*) living in their natural forest habitat. Gut bacterial composition exhibited a personal signature which became less stable as individuals aged. This lack of stability was not explained by differences in microbiota diversity but rather linked to an increase in the relative abundance of rare bacterial taxa. The lack of age-related changes in core taxa or convergence with age to a common state of the community hampered predicting gut bacterial composition of aged individuals. On the contrary, we found increasing personalization of the gut bacterial composition with age, indicating that composition in older individuals was increasingly divergent from the rest of the population. Reduced direct transmission of bacteria resulting from decreasing social activity may contribute to, but not be sufficient to explain, increasing personalization with age.

**Conclusions:**

Together, our results challenge the assumption of a constant microbiota through adult life in a wild primate. Within the limits of this study, the fact that increasing personalization of the aging microbiota is not restricted to humans suggests the underlying process to be evolved instead of provoked only by modern lifestyle of and health care for the elderly.

Video abstract

**Supplementary Information:**

The online version contains supplementary material available at 10.1186/s40168-022-01283-2.

## Background

The relevance of gut bacterial communities to host health has gained increasing interest in the last decades [1–4]. Bacterial communities of the gut contribute to maturation of the immune system, defense against pathogens, production of essential amino acids, and acquisition of energy by facilitating the digestion of milk and fibers [3, 5–8]. Imbalance, following an increase in pathogenic bacteria or loss of beneficial taxa, has been associated with a growing list of conditions, including intestinal disorders, sarcopenia, low-grade inflammation, progressive cognitive impairment, and accelerated pace of aging [6, 7, 9–11]. Taking into account age-related modifications in microbiota composition improves our understanding of the interplay between microbiota and health [9], but it remains unclear whether gut microbiota communities only contribute to or are, in turn, influenced by host aging. Indeed, age-associated changes in gut permeability [12], diet [8, 13], social relationships [14–17], deterioration of physiological systems [18, 19], especially immunosenescence [5, 6, 20, 21], could contribute to a shift in microbiota composition with age.

Clinical studies in humans shed light on the changes in gut microbiota associated with host age. Several studies highlighted a reduction in bacterial diversity in the elderly, driven by a decrease in abundant or highly prevalent core taxa in favor of rarer taxa [2, 3, 22–24]. This age-related dysbiosis [25, 26] is further characterized by an increase in pro-inflammatory and potentially pathogenic bacteria [26], and by a detrimental lack of composition stability resulting from reduced bacterial diversity [27, 28]. However, patterns assumed to be universal across humans such as decreased diversity are increasingly linked to frailty, a state of increased vulnerability to various diseases, rather than to age itself [4, 29, 30]. Disentangling the effect of age and frailty is complicated by their strong covariation because age-related changes in diet, medication, lifestyle, location, and concomitant diseases typically correlate [9, 23, 29, 31]. A second issue is the striking heterogeneity of microbiota composition between old subjects [2, 3, 9, 32], which reveals that age and frailty do not converge on a typical microbiota in terms of compositional characteristics, suggesting the existence of various paths towards an aging gut microbiota.

This heterogeneity among the elderly recently became a new focus [2] and may strongly influence therapeutic strategies [9]. If increasing interindividual variability with age results from an imbalance driven by a combination of environmental and individual factors, medical interventions aiming to “restore” a younger healthy gut microbiota profile (e.g., probiotics, microbiota transfer) will be of interest [7, 33]. On the contrary, such standardized interventions may have limited efficiency if a life-long association between a host and its microbiota shapes a personalized, stable, and healthy gut microbiota composition [11]. Individual genetics, history of diseases, and medication use could contribute towards increasing variability with age [9, 11, 34], but do not exclude more proximate drivers of variability. For example, reduced social contact associated with aging [14] could reduce the social transmission of gut bacteria [15–17, 35, 36], turning the gut of the elderly into isolated microbiota “islands.” The health consequence of this unique composition will depend on whether beneficial or pathogenic taxa are driving the pattern and its stability in the face of challenges (e.g., growth of opportunist pathogens). To date, no mechanisms explaining increasing interindividual variability with age have been proposed, and reports are limited to humans, narrowing our understanding of the origin of increased personalization with age.

Animal research on laboratory models has provided evidence for the causal influence of gut microbiota composition on host aging [10, 37]. However, laboratory animal models exhibit low genetic diversity, a gut microbiota composition strongly influenced by captivity [8, 10, 38, 39], and cannot reflect the more gradual influence of host age on gut microbial composition expected for long-lived species like humans. Wild populations of long-lived animals offer an exciting opportunity because the gut microbiota composition is likely to play a central role for the health and fitness of wild animals, who are more exposed than modern-day humans and captive animals to variation in energy resources and pathogens [40]. Recent reports suggest that natural diurnal cycles are maintained in senescent individuals [41], and that transition to old age explains little of the variation in microbiota composition between individuals [36, 40, 42–45]. However, studies mainly focused on diversity and dissimilarity of composition between subjects, while the relationship between host age, bacterial composition stability, and heterogeneity has rarely been addressed.

**Fig. 1:**
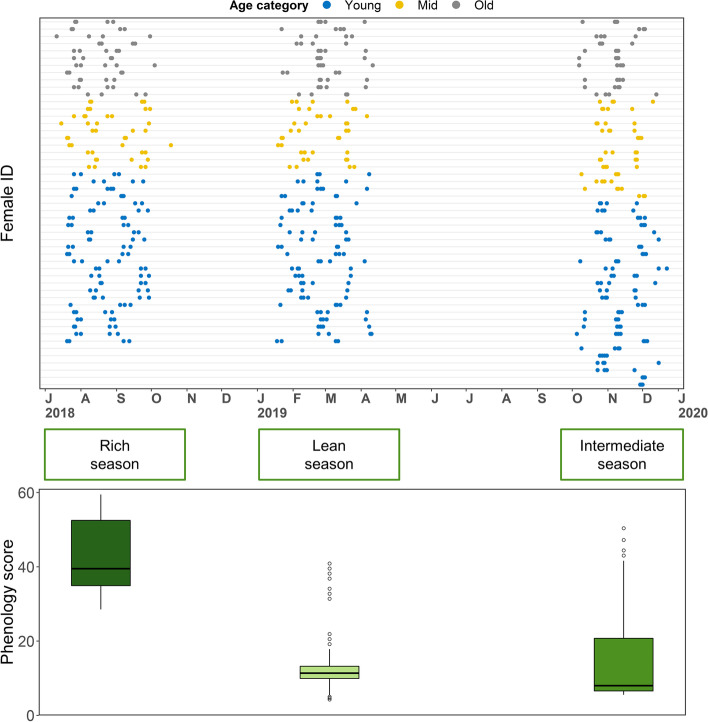
Sampling schedule with one line per subject ordered from bottom by increasing age categorized into young adult (6–10), mid-aged (> 10 and < 18), and old (≥ 18). The successive sampling windows correspond in chronological order to the rich, lean, and intermediate season, respectively. The lower panel shows food availability in the three sampling periods. Boxes represent the interquartile range (IQ), which contains the middle 50% of the records, and a line across the box indicates the median. Vertical lines extend from the upper and lower edges of the box to the highest and lowest values which are no greater than 1.5 times the IQ range. Circles represent outliers

Using a dense sampling regime on 51 adult female Assamese macaques (*Macaca assamensis*) in their natural environment over one and a half years (mean 11 ± 3 samples per individual; Fig. 1), we expand on previous investigations by testing whether gut bacterial composition changes in stability and interindividual variability with age. Macaques live in multi-male multi-female social groups, and females remain in their group of birth for their entire life, whereas males migrate between groups repeatedly. This population of wild Assamese macaques has been observed for more than a decade, with detailed records on individual life histories and behavior. First, we tested whether two features of the aging gut bacterial communities, namely (1) decreasing diversity and (2) depletion of core taxa, would be observed in a wild nonhuman primate population. Next, we investigated interindividual variability and personalization of the gut bacterial composition by testing whether (3) there is a personal signature, and (4) this signature is stable over time. We also focused on (5) age similarity as a predictor of composition similarity between individuals. Finally, we tested (6) whether the composition becomes increasingly personalized with age and (7) whether this could be explained by progressive social disengagement. We found no evidence linking age with a reduction of diversity, but the observed changes suggest an increase in rare taxa relative abundance. Most changes in composition were not consistent between individuals, so that age did not predict composition similarity. Rather, gut bacterial composition appeared personalized, decreasingly stable, and increasingly differentiated from the rest of the population as individuals aged. We discuss implications for our understanding of increasing personalization of gut microbiota composition with age in humans and other social mammals.

## Results

### Taxonomic characterization of the gut bacterial communities of adult female Assamese macaques

**Fig. 2:**
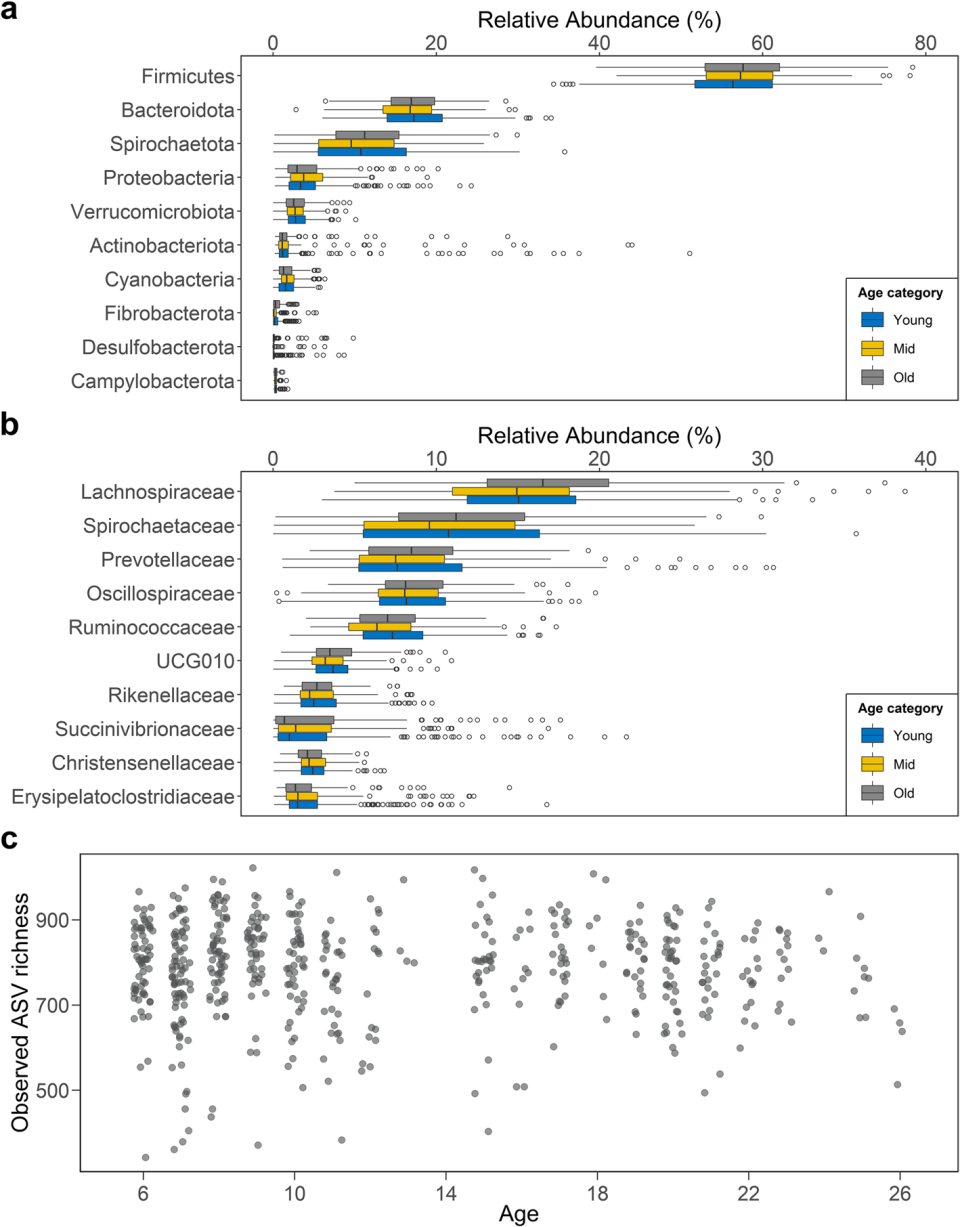
Relative abundance of the ten most abundant identified bacterial **a** phyla and **b** families in samples from female Assamese macaques representative of the adult lifespan. Boxes represent the interquartile range (IQ), which contains the middle 50% of the records, and a line across the box indicates the median. Vertical lines extend from the upper and lower edges of the box to the highest and lowest values which are no greater than 1.5 times the IQ range. Circles represent outliers. Two upper outliers in the *Firmicutes* and the Lachnospiraceae were excluded to improve displays. **c** Diversity (count of ASVs per sample) was not influenced by age in adult female Assamese macaques

Samples contained on average 785 ± 115 ASVs (range = 342–1022), and an ASV was on average found in 305 ± 161 samples (i.e., prevalence; median = 302, range = 3–543; Fig. S1-2; Tab. S1). More than 98% of the ASVs could be classified at the phylum level (11 different phyla). Those classified taxa were assigned to 37 orders (of which 36 were classified), 66 families (52 classified), and 147 genera (112 classified; Fig. 2 a–b; Tab. S2–3). Very few taxa could be assigned at the species level. The majority of ASVs belonged to the *Firmicutes* (mean per sample = 56.9%; range = 34–88%) mostly from the class Clostridia, followed by *Bacteroidota* class Bacteroidia (17.2%; 3–34%), *Spirochaetota* class Spirochaetia (11.1%; 0–36%), and *Proteobacteria* (4.4%; 0–24%) mostly Gammaproteobacteria. At the family level, Lachnospiraceae, Spirochaetaceae, Prevotellaceae, Oscillospiraceae, and Ruminococcaceae were the 5 families above 5% abundance. Members of the genus *Treponema* (10.5%) and *Prevotella* 9 (5.9%) were the two most abundant identified groups. These respective phyla, families, and genera were found in all samples (i.e., prevalence = 100%).

### Diversity of the gut bacterial community does not vary with age

To test whether decreased diversity was a feature of the aging gut bacterial communities in our study females, we investigated age-related changes in diversity with three complementary measures while controlling for cumulative rainfall in the month preceding collection, whether a female is gestating, social group, and ID. We found no evidence that observed ASV richness was predicted by any of the terms included in the models, even when considering phylogenetic relationship among taxa and evenness (full-reference comparisons likelihood ratio test: observed ASV richness, *χ*^2^ = 2.09, *Df* = 2, *p* = 0.4; FDP, *χ*^2^ = 0.42, *Df* = 2, *p* = 0.8; *H*, *χ*^2^ = 1.15, *Df* = 2, *p* = 0.6; Tab. S4 for details of full models’ estimates; Fig. 2c; Fig. S3). Results do not support our prediction that age is associated with a decrease in the diversity of gut bacterial communities.

### Relative abundance of rare bacterial taxa changes with age

Next, to test whether age was associated with evidence of dysbiosis, an increase in rare, opportunistic, or potentially pathogenic taxa, we modeled relative abundance as a function of age while controlling for season of sampling, whether a female is gestating, social group, and ID. Among phyla, only *Desulfobacteria* increased as females aged (Tab. S5), although it did not reach the significance level after correcting for multiple testing (*p* = 0.04, *FDR-p* = 0.4). This change seemed driven by an increase in an unidentified family belonging to the Bradymonadales (*p* = 0.02, *FDR-p* = 0.5), whereas no change was observed among the only other identified family from this phylum. Two of the five families in the phylum *Actinobacteriota*, the Bifidobacteriaceae and the Coriobacteriaceae, significantly decreased with age (*FDR-p* < 0.001 and *FDR-p* = 0.04, respectively). In contrast to the hypothesis of age-related dysbiosis, proportions of *Proteobacteria*, *Bacteroidetes*, and *Firmicutes* nor the *Firmicutes/Bacteroidetes* ratio (full-reference model comparison likelihood ratio test: *χ*^2^ = 1.28, *DF* = 1, *p* = 0.3) changed with age.

**Fig. 3:**
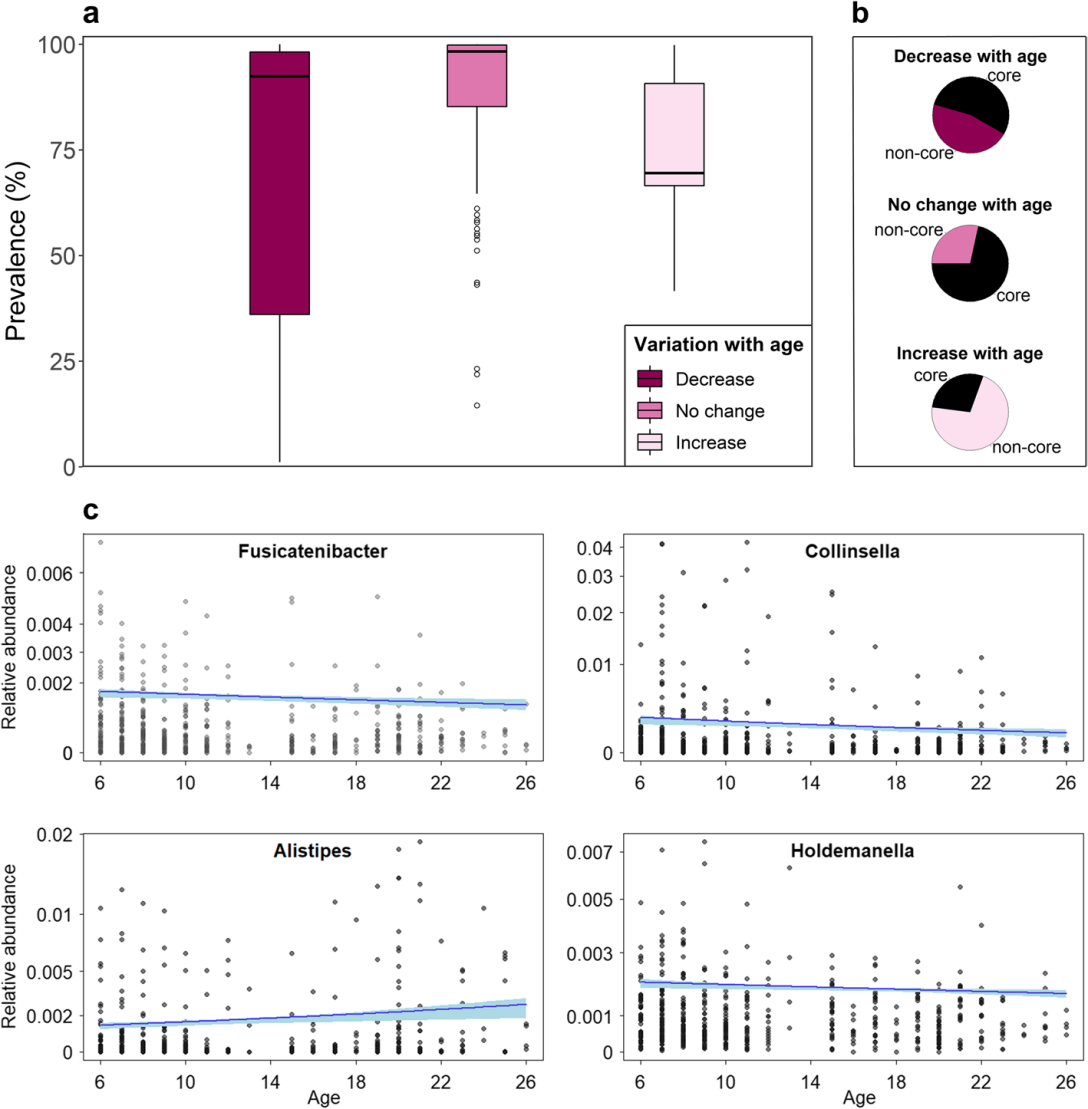
**a** Prevalence of the bacterial genera exhibiting a negative, positive, or no change in relative abundance with age. Boxes represent the interquartile range (IQ), which contains the middle 50% of the records, and a line across the box indicates the median. Vertical lines extend from the upper and lower edges of the box to the highest and lowest values which are no greater than 1.5 times the IQ range. Circles represent outliers. **b** Prevalence is qualitatively expressed as core and noncore taxa, and pie charts present the proportion of core and noncore taxa among the bacterial genera decreasing, showing no change, or increasing in relative abundance with age; core taxa are present in ≥ 90% of samples. **c** Relationship between female’s age and the relative abundance of bacterial genera associated with inflammation. The regression line and confidence interval are derived from generalized linear mixed models with beta error distribution, controlling for dummy-coded and centered social group, gestation status, and season of sampling

The relative abundance of most bacterial genera was not associated with host age (109 out of 136 genera with *p* ≥ 0.05). However, 27 genera (20% of the total number of genera) varied with age with 14 genera increasing and 13 decreasing in relative abundance as female’s age increased (*FDR-p* < 0.05 for only 13 genera equal to 9% of the genera). Age did not influence equally the core microbiota (those bacterial taxa present in at least 90% of the samples, meeting the concept of “common core” [24]; Tab. S6) compared to the noncore microbiota. Of the 14 taxa increasing with age, 10 belonged to the noncore microbiota (Fig. 3 a–b, Fig. S4). The repartition between core and noncore microbiota was even among the taxa that decreased (7 vs. 6, respectively). In comparison, most taxa that remained stable with age belonged to the core microbiota (78 out of 109 taxa). Therefore, although few genera showed consistent change in relative abundance with age across individuals, those that increased were mostly rare taxa, whereas common taxa were unaffected or decreased.

Of the 13 genera reaching significance after correcting for multiple testing (*FDR-p* < 0.05), five were from the family Lachnospiraceae (*Anaerosporobacter* and *Cellulosilyticum* increased, whereas *Eubacterium eligens*, *Lachnoclostridium*, and *Fusicatenibacter* decreased; Fig. 3c). Seven genera were distributed across several families with *Bifidobacterium*, *Ligilactobacillus*, and *Collinsella* decreasing with age, whereas *Alistipes*, Ruminococcaceae *CAG-352*, and Acholeplasmataceae *EMPG18* (matching sequences assigned to *Acholeplasma brassicae* sp. and *Acholesplasma vituli*) and Spirochaetaceae *GWE23110* increased with age (Fig. 3c). Results also suggested a decrease in the relative abundance of *Megasphaera* with age, but as this taxon was only observed in six samples (five from young or mid-aged females), this cannot be considered as a decrease in relative abundance but possibly as a presence/absence effect. Among the taxa varying with age before correcting for multiple testing (with full-reference model comparison and main effect of age being significant), we note a decrease in *Roseburia*, *Holdemanella*, *Eubacterium ventriosum*, *Enterococcus*, and *Libanicoccus* and an increase in *Eubacterium ruminantium*. The decrease in *Fusicatenibacter*, *Holdemanella*, and *Roseburia*, three genera with anti-inflammatory properties [46–50], in contrast to the increase in *Alistipes*, which includes several species with pro-inflammatory properties [51], could have consequences on host health. Among genera associated with diseases in nonhuman primates present in the dataset, namely *Campylobacter*, *Helicobacter*, *Selomonas*, *Succinivibrio*, *Streptococcus*, *Phascolarctobacterium*, and *Intestinibacter* [8, 52], only the latest increase with age. *Shigella* cannot be differentiated from other *Escherichia* by 16S rRNA gene sequencing, but *Escherichia-Shigella* did not vary with age. In conclusion, age was associated with an increase in the relative abundance of rare taxa without depletion of core taxa. Despite the absence of broad changes evocative of dysbiosis at the phylum level, noticeable changes at the genus level were consistently in favor of increased pro-inflammatory and decreased anti-inflammatory taxa in older aged females.

### Gut bacterial composition exhibits a personal signature and stability decreases with age

**Fig. 4:**
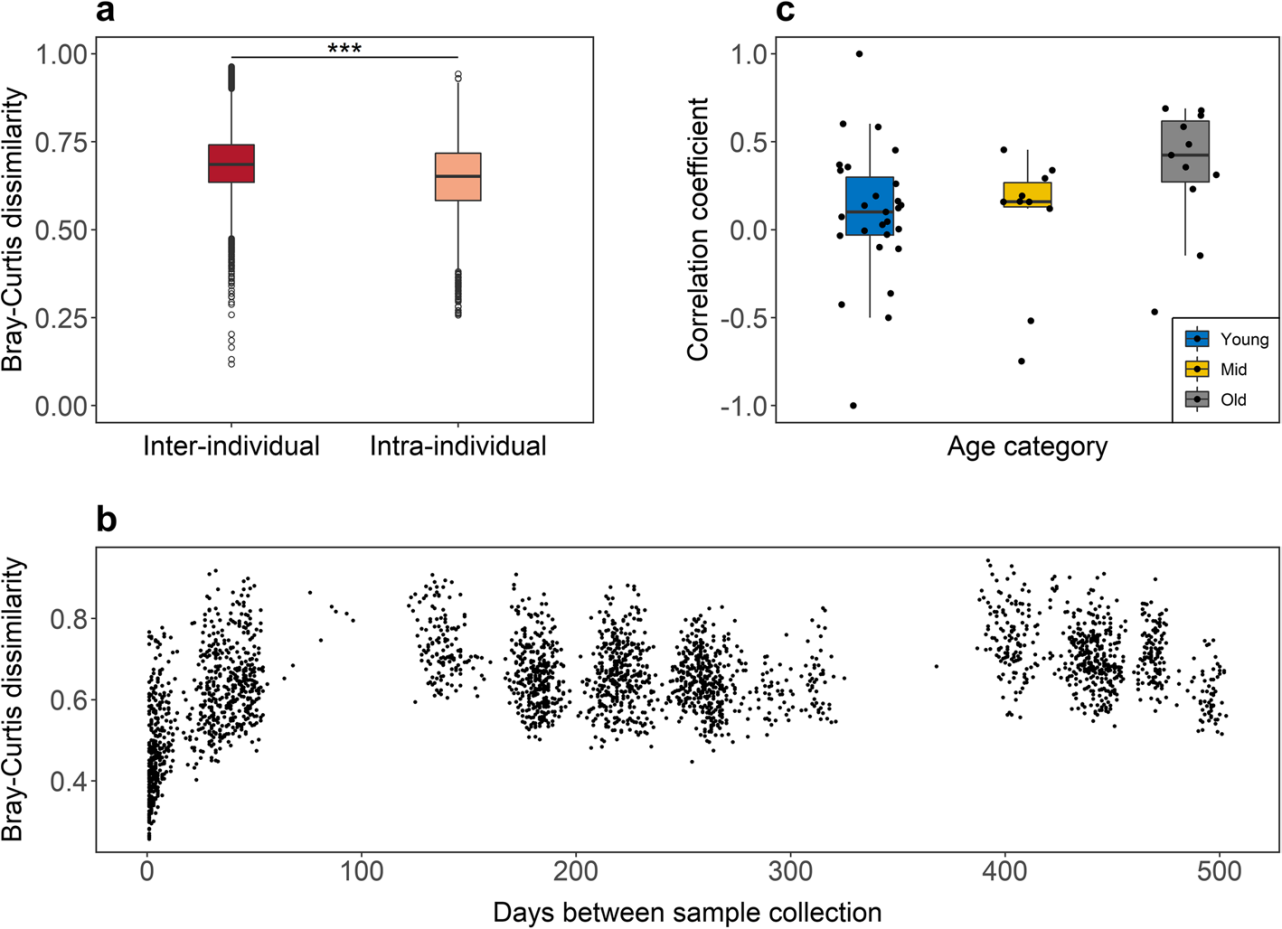
**a** Intraindividual dissimilarity in gut bacterial composition was lower than interindividual dissimilarity. **b** When all individuals were analyzed together, intraindividual dissimilarity expressed with the Bray-Curtis index increased rapidly over a few days before reaching a steady state when pairwise sample dissimilarity did not increase anymore, even for samples collected more than a year apart. **c** Composition stability expressed as the correlation coefficient between intraindividual dissimilarity and time gap between sample collection (measured in days). Female’s stability coefficients are depicted for young adult (6–10), mid-aged (> 10 and < 18), and old (≥ 18) females (boxes and whiskers), with more positive values indicating lower stability. Boxes represent the interquartile range (IQ), which contains the middle 50% of the records, and a line across the box indicates the median. Vertical lines extend from the upper and lower edges of the box to the highest and lowest values which are no greater than 1.5 times the IQ range. Circles represent outliers

Individual identity explained the largest portion of composition dissimilarity (Tab. 1), with lower intra- than interindividual dissimilarity (mean ± SD of intraindividual vs. interindividual BC, 0.64 ± 0.11 vs. 0.69 ± 0.08, *r* = 0.05, *p* < 0.001; Wunifrac, 0.29 ± 0.09 vs. 0.30 ± 0.09, *r* = 0.01, *p* < 0.001; UWunifrac, 0.31 ± 0.08 vs. 0.33 ± 0.07, *r* = 0.02, *p* < 0.001: Fig. 4 a–b; Tab. S7). The effect translates into an average 3–8% lower intra- compared to interindividual dissimilarity. We predicted that if gut bacterial composition gradually changes over time, samples collected from the same individual further apart in time would display greater dissimilarity than samples collected within a short period of time. We found a significant positive association between dissimilarity and time gap between collection dates (*n* = 51 females; BC, *r* = 0.38, *p* = 0.002; Wunifrac, *r* = 0.23, *p* = 0.06; UWunifrac, *r* = 0.30, *p* = 0.01). Yet upon closer examination of the data, pairwise dissimilarity sharply increased in a 10-day period, before reaching a plateau. After removing pairwise dissimilarity values from samples collected up to 10 days apart (which removed 3 females from the dataset), to test for a gradual modification of the gut bacterial composition over a longer time, no association remained (*n* = 48 females; BC, *r* = 0.18, *p* = 0.7; Wunifrac, *r* = 0.04, *p* = 0.9; UWunifrac, *r* = 0.08, *p* = 0.9; Tab. S7). In other words, when all individuals were analyzed together, the rate of gut bacterial change over time appears rapid before reaching a steady state, possibly as the result of core taxa being retained throughout the year (Fig. 4b). However, the change of dissimilarity over time differed depending on the age of the individual. Correlation coefficients between dissimilarity of two samples from the same individual and time elapsed between sampling events were much higher in old than in mid-aged and young adult females (*n* = 48 females, Kruskal-Wallis = 6.04, *DF* = 2, *p* = 0.049); stability of gut bacterial composition decreased with age. Despite previous clinical reports on a relationship between composition diversity and stability [27, 28], gut bacterial diversity did not predict composition stability, and therefore did not explain the reduced stability with age in this wild population (details in supplementary material; Tab. S8; Fig. S5).

**Table 1:** Effect sizes of predictors of gut bacterial composition dissimilarity

		*R*^2^ (%)		
Predictor	Df	BC	Wunifrac	UWunifrac
ID^1^	50	19.60	14.34	18.00
Season^2^	2	6.78	7.03	6.81
Group^2^	2	3.24	1.77	2.59
Age^2^	1	0.52	0.38	0.55
Gestation^2^	1	0.27	0.16	0.35

*p*-values derived from 10,000 permutations. All *p* < 0.001 except for gestation (all *p* > 0.1). ^1^Model including only ID as a predictor. ^2^Model including ID as strata to account for repeated sampling of the individuals

### Age is not a strong predictor of gut bacterial composition

**Fig. 5:**
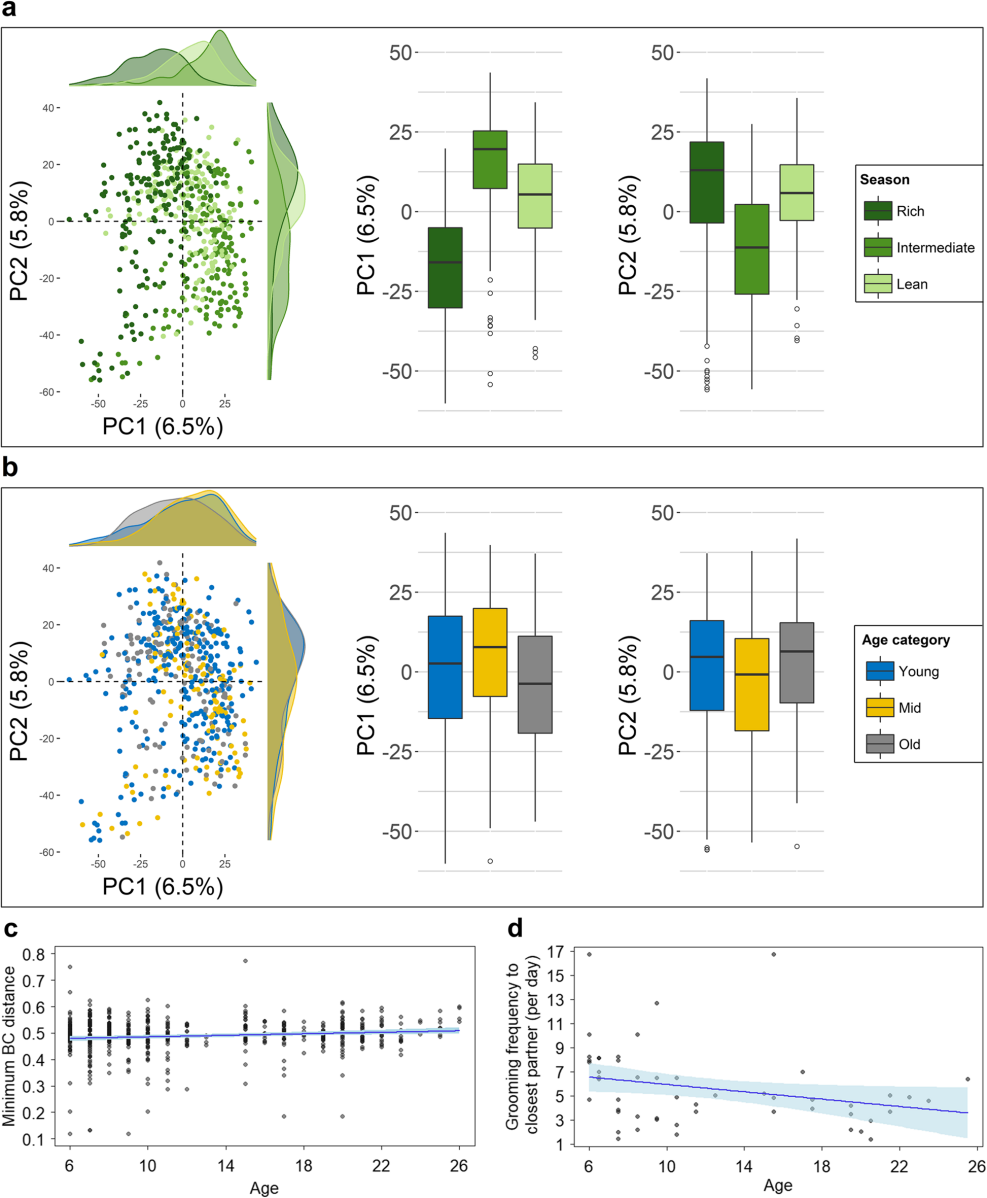
**a** and **b** Principal component analysis of BC dissimilarity between gut bacterial compositions of adult female Assamese macaques. Dissimilarity was influenced by **a** season (classified by food abundance) but not **b** age categorized into young adult (6–10), mid-aged (> 10 and < 18), and old (≥ 18). Boxes represent the interquartile range (IQ), which contains the middle 50% of the records, and a line across the box indicates the median. Vertical lines extend from the upper and lower edges of the box to the highest and lowest values which are no greater than 1.5 times the IQ range. Circles represent outliers. One outlier was excluded to improve displays. **c** Personalization of gut bacterial communities increased with age. Personalization is expressed per sample as the minimum dissimilarity to any sample from another female in the same group and season. **d** The older females were the less often they groomed with their closest female partner. Regression lines and confidence intervals are derived from **c** a generalized linear mixed model with beta error distribution and controlling for gestation status, social group, and season of sampling or **d** from multiple linear regressions with Gaussian distribution, controlling for social group

Next, we tested the influence of several predictors on the variation in the entire gut bacterial community composition, summarized in dissimilarity metrics (BC, WUnifrac, UWUnifrac), using a PERMANOVA analysis. After accounting for the effect size of ID on composition dissimilarity, season of sampling explained the largest proportion of variation, followed by social group (Tab. 1). Whether the female was gestating or not did not influence composition. Age categorized into young adult, middle aged, and old had a minor influence on bacterial composition. To illustrate, PCA components 1 and 2 do not differ between age categories but only between seasons (Fig. 5 a–b). If age entered the PERMANOVA as a continuous predictor, it again did not explain much of composition (below 1% variance explained, Tab. 1). In other words, the age of a female could not be deduced from her gut bacterial composition. This suggests that most of the variation observed with age was not consistent across females but may be rather unique to each individual.

### Personalization of gut bacterial communities increases with age

We tested whether the absence of consistent changes in gut bacterial composition could be explained by the tendency of older females to display a unique gut bacterial composition. Results tended to support the hypothesis that personalization was positively associated with age (Tab. 2). Personalization, measured as minimum interindividual dissimilarity, increased from 0.48 to 0.51 from the youngest to the oldest age (6–26 years in this study population) (Fig. 5c; Tab. 2). Personalization was also greater in the rich season, possibly in interaction with age, again indicating increasing personalization with age. In the absence of detailed feeding data, the mechanism exacerbating personalization in the rich season must remain unknown. Furthermore, the variance in minimum dissimilarity decreased with age, which suggest that old females displayed more consistently increased minimum dissimilarity than younger females. Although this tended to strengthen the positive association between age and personalization, this greater dispersion in young females was not predicted a priori and should be interpreted with caution. These effects were revealed by the models based on BC (full-reference model comparison likelihood ratio test: *χ*^2^ = 15.25, *df* = 5, *p* = 0.009) and Wunifrac (*χ*^2^ = 23.40, *df* = 5, *p* < 0.001), whereas the model on UWnifrac did not perform significantly better than the reference model (*χ*^2^ = 7.55, *df* = 5, *p* = 0.2; Tab. S9). BC and Wunifrac account for the relative abundance of ASVs, whereas UWnifrac only considers their presence or absence. Therefore, results reveal that increasing personalization with age arose from a shift in the relative abundance rather than in differences in presence/absence of the bacteria composing the gut microbiota.

**Table 2:** Result of the models for gut bacterial composition personalization. Personalization expressed as minimum dissimilarity Bray-Curtis (BC) and weighted unifrac (Wunifrac) (*n* = 543 samples). Season is marginally significant in one model and significant in interaction with age in the other

	Fixed effects	Levels	Estimate	***SE***	***CI***_**lower**_	***CI***_upper_	***p***
**BC**	Intercept		−0.04	0.04	−0.11	0.03	-
	**Age**^**1**^		**0.03**	**0.01**	**0.01**	**0.06**	**0.02**
	Season^2^						0.06
		M (ref L)	0.03	0.04	−0.05	0.09	
		R (ref L)	−0.06	0.04	−0.12	0.01	
	Group^3^						0.5
		MST (ref MOT)	0.03	0.03	−0.02	0.09	
		SST (ref MOT)	0.04	0.04	−0.04	0.11	
	**Gestation**		−**0.08**	**0.04**	−**0.15**	−**0.00**	**0.05**
Dispersion parameter	Intercept		3.98	0.07	3.87	4.12	-
	**Age**^**1**^		**0.38**	**0.07**	**0.25**	**0.50**	**< 0.001**
**Wunifrac**	Intercept		−1.68	0.03	−1.74	−1.62	-
	Age^1^		−0.02	0.02	−0.05	0.02	-
	Season^2^						-
		M (ref L)	0.11	0.03	0.05	0.18	
		R (ref L)	0.06	0.03	0.00	0.11	
	Group^3^						0.6
		MST (ref MOT)	0.01	0.02	−0.03	0.05	
		SST (ref MOT)	0.03	0.03	−0.02	0.08	
	Gestation^4^		−0.05	0.03	−0.11	0.01	0.08
	**Age**^**1**^ **×** **season**^**2**^						**0.04**
		**Age × M (ref L)**	**0.01**	**0.03**	−**0.04**	**0.07**	
		**Age × R (ref L)**	**0.06**	**0.02**	**0.02**	**0.10**	
Dispersion parameter	Intercept		4.99	0.07	4.89	5.15	
	**Age**^**1**^		**0.28**	**0.07**	**0.17**	**0.43**	**< 0.001**

^1^Z-transformed to a mean of 0 and sd of 1; mean and sd of age on the original scale are 12.44 and 5.79 years, respectively. ^2^Categorical predictor with 3 levels: lean, intermediate, and rich, with the reference level set to the lean season. ^3^Categorical predictor with 3 levels MOT, MST, SST, with the reference level sets to group MOT. ^4^Binary predictor with reference sets to non-gestating

### Progressive decreasing social activity with age as a possible driver of gut bacteria personalization

Our final aim was to test whether social interactions between individuals promoted bacterial transmission which could tend to homogenize bacterial composition between dyads. Dyads grooming more frequently shared slightly closer bacterial composition (*β* = −0.01, 90 *CI*_lower_ = −0.02, 90 *CI*_upper_ = 0.00, *p*− = 0.95), an effect also captured, but with much less certainty, by the duration of interaction (*β* = −0.01, 90 *CI*_lower_ = −0.01, 90 *CI*_upper_ = 0.00, *p*− = 0.87; Tab. S10). This suggests that social transmission occurred but had a small influence on gut bacterial dissimilarity between individuals, with an average 2% decrease in dissimilarity from dyads belonging to the highest percentile of the grooming frequency distribution compared to dyads in the lowest percentile.

From these results, we asked whether increasing personalization was associated with a tendency of old females to be socially less active, which would limit social transmission of gut bacteria. As females aged, and as predicted from literature, frequency of grooming interactions with the closest (top) social partner decreased (*F*_3,47_ = 5.917, *p* = 0.002; age, estimate ± SE = −0.27 ± 0.13, *p* = 0.04; Fig. 5d; Tab. S11A) as did the average grooming frequency with all females in the social group (*F*_3,47_ = 11.79, *p* < 0.001; age, estimate ± *SE* = −0.27 ± 0.11, *p* = 0.02; Tab. S11B). Using the duration of grooming instead of frequency produced similar results (Tab. S11 C–D). Yet, introducing female’s top and average grooming frequency in models testing gut bacterial personalization (see “Personalization of gut bacterial communities increases with age” above) did not influence the relationship between age and personalization, and neither top partner nor average grooming frequency was significantly associated with personalization (Tab. S12). Therefore, despite evidence for a decrease in social grooming interactions with age, there is no evidence that increasing gut bacterial personalization with age is primarily driven by a reduction in close contact interactions with a female’s partners, possibly due to the weak link observed between social contact and gut bacterial composition in the population.

## Discussion

Our findings are consistent with the hypothesis that age is associated with modifications in the composition and dynamics of the gut bacterial microbiota over the adult lifespan. Specifically, trajectories observed in wild female macaques revealed increasingly personalized gut bacterial composition with age, a pattern thought to be restricted to humans and attributed to pronounced differences in lifestyle, medication, and concomitant diseases among the elderly [1–3, 29, 31]. Gut bacterial communities of the study females carried a strong personal signature, whose stability decayed over time in old individuals possibly linked to an increase in the relative abundance of rare bacterial taxa with age. Bacterial diversity could not explain this lack of stability, first because age was not associated with a decrease in diversity and second because there was no evidence for a causal relationship between gut bacterial diversity and stability. Finally, reduced direct transmission of bacteria resulting from decreasing social activity may contribute to, but not be sufficient to explain, increasing personalization with age.

Host identity arises as a strong predictor of microbiota composition [15, 32, 40, 41, 43, 45, 53–57], diversity, and even stability [11, 41, 54, 58, 59] in both humans and nonhuman animals. In our study population, individual identity explained the largest part of the variance in gut bacterial composition, and samples collected from the same host were more similar than samples from different hosts. The small effect size of the intra- vs. interindividual comparison is consistent with previous results showing that personal signature is less obvious when samples collected from the same individuals long apart in time are compared with samples collected in the same period from different individuals [35, 43].

We propose two possible mechanisms leading to increasing personalization with age. First, it could be that the results of a lifetime of individualized selection pressures on hosts shape increasingly personal microbiota. For example in baboons, host’s genetic contributions to microbiota composition increase with age [34]. Such personalization should translate in a stable microbiota composition and could be associated with healthier aging for the host. In support, microbiota personalization increased with age and was associated with healthier levels of serum markers and higher survival in a large human cohort study [11]. Under this scenario, a microbiota composition tailored to the host’s way of living could help “make the most of an aging body” [1, 57, 60]. As different bacterial taxa may possess similar functions [11, 61, 62], sequencing metagenomes of the gut communities will be needed to investigate how personalization may influence services provided to the host. Alternatively, it could be stochastic changes in the microbiota composition resulting from dysbiosis that cause gut microbiota of older individuals to appear more personalized. This could translate in a less stable composition characterized by more frequent and larger changes in taxa relative abundance, possibly associated with an increase of rare taxa in older compared to younger subjects. We review our results in the light of these two possible scenarios.

The rapid increase in intraindividual composition dissimilarity in our study aligns with findings in humans showing that samples collected a week apart were not more similar than those collected 3 months apart [58] rather than with evidence of a constant signature [56]. In humans, antibiotic medication may be a prominent cause of bacterial turnover [58, 63], whereas in wild nonhuman primates, natural environmental factors may cause the rapid decrease in individual microbiota signature [43, 59]. For example, season had the second most important influence on composition in our subjects, reflecting the previously established influence of diet, rainfall, or circadian rhythms on the microbiota [24, 40]. Although microbiota stability is influenced by the environment, it may also differ between individuals [27, 54, 58, 63], with lower stability linked to worsened health outcomes [27]. Here, intraindividual stability was lower in older subjects which aligns with personalization stemming from dysbiosis. In elderly humans, microbiota composition is stable over a couple of months [32], but more recent evidence suggests stability may decrease with age when samples are collected years apart, although the effect did not reach significance [27]. This study is the first to investigate the relationship between host age and microbiota stability in species other than humans and their ape closest relatives.

In several clinical studies, gut bacterial diversity at one time was associated with stability to a subsequent period, which has been interpreted as diversity promoting health [27, 28, 58, 63]. Our results suggest that great care is needed in analyzing this relationship because the temporal correlation between diversity and stability indicating the causal relationship can be reversed. In this and other studies, bacterial diversity did not change with age [24, 40, 42], suggesting that the decline observed in humans and captive animals may not be a biological inevitability but rather associated with lifestyle. Finally, poor stability may not be linked to low diversity but rather to the increase in the relative abundance of rare opportunist taxa [27, 53, 64].

The consequences of an increase in rare taxa for host health is debated. It is linked to the notion of dysbiosis, when imbalance and depletion in the core bacterial communities allow rare taxa to proliferate, often deemed to have negative consequences [61]. Thus, the increase in rare taxa relative abundance combined with higher composition turnover with age in wild macaques could suggest age-related dysbiosis. However, rare taxa may promote extreme aging [22] by replacing core members and producing important metabolites essential to a senescent organism [11]. Services provided by the gut bacterial communities may become especially critical when old subjects face inflammaging [65]. In this context, taxa with anti-inflammatory properties and producing short-chain fatty acids (SCFAs) may contribute to healthy aging [66]. As seen in humans and captive macaques [22, 65, 67], we found a decrease in *Fusicatenibacter*, *Holdemanella*, *Roseburia*, and some members of the genus *Eubacterium*, all known for their anti-inflammatory or SCFAs producing properties [46–49, 66]. Those changes not only echo but may contribute to the increased inflammatory immune profile, or be promoted by senescent macrophages activity, observed in aging macaques [20, 68]. At the same time, and also in accordance with changes observed in captive macaques [67], Ruminococcaceae (CAG-352) and other members of *Eubacterium* increased in abundance with age. These concomitant increase and decrease illustrate the difficulties to draw conclusions on resulting anti-inflammatory properties of the gut microbiota of individuals of different ages based on 16S rRNA gene sequencing. Rather than extrapolating health consequences of gut bacteria from laboratory studies to wildlife, insights could be drawn from linking changes in bacterial abundance associated with age and those resulting from challenges relevant to the species under study (e.g., adverse climatic events, social and physiological adversity).

Understanding the consequences of microbiota dynamics on host health has strongly relied on the search for age-related changes in the abundance or presence of specific taxa. Thus, *Alistipes* increased with age in wild macaques, echoing its increased relative abundance in the elderly [32, 65]. Nevertheless, identifying consistent changes with age across studies and species remains difficult [2], and some of our results contrast with dynamics observed in other macaques [42, 67, 69, 70]. For example, captive macaques showed some parallels to humans, including the decrease of *Firmicutes/Bacteroidetes* ratio in old subjects [67, 69], although findings were, like in the present study, not always robust to corrections for multiple testing nor confirmed in our and another free-ranging macaque population [42]. The opposite age trends reported for the health-associated *Bifidobacterium* in this study versus a previous report on another macaque species [42] are representative of the conflicting results also reported in humans [2, 22]. Also, we found a decrease, not an increase like Wei et al. [67], in *Megasphaera* responsible for gastrointestinal disorders in primates [52]. The mucin degraders *Akkermansia*, scrutinized for their association to extreme longevity [2, 22] and increasing with age in wild lemurs [71], were isolated from infants in our study populations (data not shown), but not in adults. Importantly, the number of taxa associated with age appears much lower when comparing wild to captive primates [40, 42, 67, 70, 71], which questions the insights gathered from each model. Beyond variation in a few taxa, the description of broader patterns offers a wider framework to test and refine.

Social animals acquire gut microbiota from conspecifics [17] which translates into greater homogeneity within than between social groups [16, 36, 40, 43], also detected here. In humans, the size of an individual’s social network decreases with age, either due to the loss of similar-aged partners or to increasing social selectivity [14]. Evidence of similar decreasing social engagement has been gathered from several nonhuman primate species [72]. Therefore, we postulated that increasing personalization may result from decreasing social activity of older individuals. Although we found that social interaction rates decrease as females aged, no direct link between engagement in social interactions and gut bacteria personalization could be found. Failure to detect a social contact effect may result from our treatment of behavioral data for the analysis or from general activity decline overriding the social contact effect.

Both the overall weak effect size attributed to social transmission on bacterial composition in the population and the lack of a social contact effect on personalization may result from dyadic social interaction scores being calculated over the entire 1.5 years study period. This analysis was chosen to avoid biases associated with scarce records of interaction in wild animals [73] but came at the expense of precision in matching time windows of social interactions concurrent to the gut bacterial composition [15]. Alternatively, the general decline in activity above and beyond social activity may have driven the increase in microbiota personalization with age if aging individuals each sample a smaller and different portions of the environment when they rest more, feed less, and roam less. Furthermore, the physiological regulation of overall activity levels via hormones may affect the gut microbiota [74, 75] in more profound ways than a decline in social transmission of microbiota.

Our results also contribute to a more complete macaque model of senescence [68]. The gradual and linear increase in personalization of the gut bacterial composition parallels the continuous decline in female macaque fertility [76]. Gut bacterial composition appeared stable in young and mid-aged females, but less so in the oldest group, which echoes the late onset around 18 years of age in loss of body mass [20], muscle mass [77], eyesight [78], and bone mineral density [79]. Our investigation of gut bacterial and social age-related changes was a first attempt to put senescence in perspective across different physiological systems. This is necessary to identify plausible drivers (e.g., tooth wear, immunosenescence) and fitness consequences (e.g., muscle loss, declining energy balance constraining fertility) of age-related changes in gut bacterial composition. The possibility that gut bacterial composition exhibits terminal senescence [80], independent of age, has not been investigated in this or previous studies on mammals due to lack of data on individual survival and remains open

## Conclusion

Our study adds to a growing body of data elucidating the relationship between age and gut microbiota in nonhumans. Research in wild animal systems is essential to disentangle phenomena rooted in our long shared evolutionary history from those driven by humans’ contemporary ways of living. The transition from an immature to an adult microbiota, the personal signature attached to microbiota composition, and now possibly also its increasing personalization with age are among the former. On the contrary, decreasing microbiota diversity with age is mostly reported in humans and captive animals, incriminating our humanized environment. The fact that personalization is observed in wild systems, despite flow of bacterial taxa between populations during migration [43, 73] and social transmission through frequent body contact [16, 17], suggests that strong and conserved mechanisms are at play. Understanding the processes underlying this increasing personalization with age, its possible association with instability of the microbiota and inflammaging, is therefore critical to predict consequences for, and act on, host health.

## Methods

### Study site and study subjects

This research was conducted between July 2018 and December 2019, with an interruption from August to October 2019, in the Phu Khieo Wildlife Sanctuary (PKWS, 16° 05′–35′ N, 101° 20′–55′ E, 1573 km^2^), which is part of the Western Issan Forest Complex in northeastern Thailand [81], a contiguous protected forest area of > 6500 km^2^. Within the PKWS, the long-term study site Huai Mai Sot Yai (16° 27′ N, 101° 38′ E, 600–800 m above sea level) consists mostly of dry evergreen forest and bamboo stands at a mean annual temperature of 21.2 °C [81, 82], with a clearly differentiated dry season from November to March and a rainy season from April to October [81, 82].

We collected data from three social groups of fully habituated and individually recognizable Assamese macaques from the morning to the evening sleep tree (from about 5 am to 6 pm). Assamese macaques are primarily arboreal and typically occupy a 4 km^2^ home range [82]. Each study group was composed of several adult females (*n* = 9–23), their immature offspring, and several adult and subadult males (*n* = 6–20) for a total group size between 41 and 97, with fluctuations within groups following births, emigrations, and disappearance of group members, and immigration of males. Females were considered adult from the mating season of first conception (usually at 5.5 years of age), and ages were either known from exact date of birth within days or weeks or inferred by experienced members of the research team based on morphological comparison with individuals of exactly known age. Assamese macaques are highly seasonal breeders with 79% of births occurring in a 3-month period (JO and OS unpublished data), so female ages were expressed in years ranging between 6 and 26. When necessary for graphical representation, ages were categorized as young adult (6–10), mid-aged (> 10 and < 18), and old (≥ 18) (Fig. 1c) based on the time at onset and acceleration of physical signs of senescence in macaques [77, 83].

### Social behavior collection

Social behaviors were recorded during 40 min continuous focal animal protocols [84] on all 51 adult females in the three study groups, resulting in a total of 2729 observation hours (53.5 ± 17.9 h per female). Frequency and duration of affiliative grooming interactions along with the identity of the giver and receiver were recorded. Individuals were in body contact during grooming which involves hand and mouth contact with a conspecific’s fur, which may promote transfer of microbiota between individuals [17].

### Fecal sample collection

Fecal samples were collected during focal animal protocols and opportunistically immediately upon defecation in the field. The identity of the individual and date and time of defecation were recorded. Samples were homogenized, and ca. 500 mg was placed in 1 ml RNAlater buffer (ThermoFisher https://www.thermofisher.com/content/dam/LifeTech/migration/en/filelibrary/nucleic-acid-purification-analysis/pdfs.par.18819.file.dat/bp-7020.pdf), shaken for ca. 1 min, and protected from sunlight in the field bag. Samples were incubated for a minimum of 24 h away from sunlight at room temperature before they were frozen at −20 °C until transportation on dry ice to Germany where they were kept at −20 °C or −80 °C.

For this study, we selected a subset of 543 fecal samples collected from the 51 females in three seasons defined according to fruit availability (mean ± SD = 11 ± 3 samples per individual, median = 12, range = 3–13; mean = 4 ± 0.4 samples per individual per season, range = 2–5) (Fig. 1 a–b; see also supplementary material for details on fruit phenology scores). For each sample, we calculated the cumulative rainfall in mm in the preceding 30 days from satellite recordings by the Tropical Rainfall Measuring Mission available through the Goddard Earth Sciences Data and Information Services Center [85].

### DNA extraction, amplification of 16S rRNA genes, and sequencing

After thawing samples on ice, the RNAlater buffer was removed by centrifuging samples 3 min at 4000 rpm on a Thermo Electron Corp Heraeus Pico 21 (ThermoFisher Scientific). DNA was extracted from 150 mg of fecal matter with a DNeasy PowerSoil Pro Kit (QIAGEN, Cat. No./ID: 47016) following manufacturer instructions. The quality of DNA extraction was assessed by spectrophotometry on a NanoDrop ND-1000 spectrophotometer (ThermoFisher Scientific) by visually inspecting the shape of the curve and peak at 260-nm wavelength. Samples yielding a DNA concentration lower than 6 ng/μl were discarded or extracted once more, when possible, but if again too low eventually discarded. DNA was diluted to a final concentration of 10 ng/μl, and then the V3-V4 region of the 16S rRNA gene was amplified using PCR primers as described by Klindworth et al. [86]. Primers included adapters for MiSeq sequencing (underlined, forward primer: S-D-Bact-0341-b-S-17 5′-TCGTCGGCAGCGTCAGATGTGTATAAGAGACAG-CCTACGGGNGGCWGCAG-3′, reverse primer: S-D-Bact-0785-a-A-21 5′-GTCTCGTGGGCTCGGAGATGTGTATAAGAGACAG-GACTACHVGGGTATCTAATCC-3′). Each PCR contained 10 μl of fivefold Phusion GC buffer, 0.2 μl 50 mM MgCl_2_, 2.5 μl 5% DMSO, 1 μl 10 mM dNTPs, 31.3 μl nuclease-free water (Ambion), 1 μl of forward and 1 μl of reverse primers (equivalent to 0.2 mM), 0.5 μl of Phusion high-fidelity DNA polymerase (2 U/μl; ThermoFisher Scientific), and 2.5 μl of 10 ng/μl DNA extract for a total volume of 50 μl. PCR were performed in triplicate on a Labcycler Basic (SensoQuest) with an initial denaturation at 98 °C, followed by 25 cycles of denaturation at 98 °C for 45 s, annealing at 55 °C for 45 s, and elongation at 72 °C for 30 s. The final elongation was at 72 °C for 5 min, and samples were then maintained at 10 °C until further processing. Amplification efficiency and purity were confirmed by visualizing PCR products by agarose gel electrophoresis and by inclusion of negative and positive controls on all runs.

Amplicon triplicates were pooled, and PCR products were used to attach indices and Illumina sequencing adapters using the Nextera XT Index kit (Illumina, San Diego) and the KAPA HiFi HotStart ReadyMix (Roche Diagnostics, Mannheim, Germany). Index PCR was performed using 5 μl of template PCR product, 2.5 μl of each index primer, 12.5 μl of 2x KAPA HiFi HotStart ReadyMix, and 2.5 μl PCR grade water. Thermal cycling scheme was 95 °C for 3 min, 8 cycles of 30 s at 95 °C, 30 s at 55 °C and 30 s at 72 °C, and a final extension at 72 °C for 5 min. Products were quantified using the Quant-iT dsDNA HS assay kit and a Qubit fluorometer (Invitrogen GmbH, Karlsruhe, Germany) following manufacturer’s instructions. Purification of the indexed products was performed using MagSi-NGSPREP Plus magnetic beads (Steinbrenner Laborsysteme GmbH, Wiesenbach, Germany) as recommended by the manufacturer, and normalization was performed with the JANUS Automated Workstation from Perkin Elmer (Perkin Elmer, Waltham Massachusetts, USA). Sequencing was conducted using Illumina MiSeq platform using dual indexing and MiSeq reagent kit v3 (600 cycles) as recommended by the manufacturer. Five samples were extracted, amplified, and sequenced in triplicates giving a total of 15 replicates, which revealed that differences in bacterial composition arising from lab procedure are an order of magnitude lower than real biological differences between samples (Fig. S6).

### 16S rRNA gene sequence data deposition

The raw sequence data from the 16S rRNA gene amplicons were deposited at the National Center for Biotechnology Information and can be assessed under the BioProject accession number PRJNA795139.

### Sequence processing, taxonomic assignment, and dataset preparation

Raw paired-end sequences were quality-filtered using fastp v0.20.0 [87] with a minimum phred score of 20, minimum sequence length of 50 bases and sliding window size of 4, read correction by overlap, and adapter removal of the sequencing primers. Quality-filtered reads were merged with PEAR v0.9.11 [88]; 16S rRNA gene primers were trimmed with cutadapt v2.5 [89]. VSEARCH v2.15.0 [90] was used to filter the sequences by size (min length ≥ 300 bp), remove duplicates (--derep_fulllength), and remove erroneous sequences (--cluster_unoise, UNOISE3 with default settings [91], following recommendations www.drive5.com/usearch/manual/cmd_unoise3.html). We further performed de novo chimera removal (--uchime3_denovo) followed by referenced_based chimera removal (--uchime_ref) against the SILVA SSU 138.1 NR database [92]. Finally, sequences were mapped to amplicon sequence variants (ASVs) with VSEARCH (--usearch_global) with default sequence identity threshold of 0.97. We assigned taxonomic classification to ASVs using Bayesian-based lowest common ancestor (BLCA) algorithm [93] (version 2.1) against the SILVA SSU 138.1 NR database at the default 90% identity threshold through BLAST (version 2.9.0+). ASVs comprising extrinsic domains, eukaryotes and archaea, were removed from the ASV table using amp_subset_taxa. A total of 23,206,649 reads corresponding to 5375 ASVs were obtained from the 543 samples, with all samples achieving high read counts (mean ± SD = 42,738 ± 24,849 reads per sample, median = 36,340, range = 10,839–150,307). Finally, we applied a threshold of 0.25% on the relative abundance to filter spurious ASVs [94]. The final dataset included 1399 ASVs (Tab. S1; Fig. S7). No general agreement exists on the handling of spurious ASV sequences, and filtering efficiently reduces false-discovery rate at the expense of statistical power [94, 95]. To test the robustness of the findings to the inclusion or exclusion of spurious ASVs, analyses were repeated on the full and the filtered datasets (including 5375 and 1399 ASVs, respectively). Results reported in the main text on the conservative filtered dataset agreed with conclusions drawn on the full dataset (Tab. S13–15).

### Statistical analyses

Analyses were performed with a sample size of 543 samples from 51 females, unless specified otherwise. Descriptive statistics, calculation of diversity metrics and analyses, bar plots, and graphs were done with R (R Core Team, 2020; version 4.0.2) through the RStudio interface (version 1.4.1106) using the packages *ampvis2* (v.2.7.5), *ape* (v.5.5), *brms* (v.2.16.1), *car* (v.3.0–11), *cowplot* (v.1.1.1), *ggExtra* (v.0.9), *ggsci* (v.2.9), *ggpubr* (v.0.4.0), *glmmTMB* (v.1.1.2.2), *gridExtra* (v.2.3), *GUnifrac* (v.1.3), *janitor* (v.2.1.0), *lme4* (v.1.1–27.1), *lmerTest* (v.3.1–3), *lubridate* (v.1.7.10), *picante* (v.1.8.2), *phangorn* (v.2.7.1), *plyr* (v.1.8.6), *reshape2* (v.1.4.4), *tidyverse* (v.1.3.1), and *vegan* (v.2.5–7) (referenced in supplementary material). A phylogenetic tree of ASVs was generated by aligning all sequences of the filtered dataset with MAFFT v7.407 [96] at a maximum of 100 iterations. The tree was calculated using FastTree 2.1.10 (OpenMP) [97], saved in newick format and midpoint rooted using FigTree (version 1.4.4) (https://github.com/rambaut/figtree/). For linear models, continuous predictors were z-transformed (mean of 0, standard deviation of 1), categorical predictors dummy coded as fixed terms and centered-dummy coded when included in random slopes. Two-sided *p*-values < 0.05 were deemed significant. In graphical representation using boxplots, boxes represent the interquartile range (IQ), which contains the middle 50% of the records. Vertical lines extend from the upper and lower edges of the box to the highest and lowest values which are no greater than 1.5 times the IQ range, and a line across the box indicates the median. Circles represent outliers, whereas datapoints are depicted by dots.

### Diversity of gut bacterial communities and age

To estimate sample diversity, we computed three complementary alpha-diversity measures: the observed ASV richness (count of ASVs per sample, amp_alphadiv function in *ampvis2* relying on the diversity function from *vegan*), Faith’s phylogenetic diversity (FPD, function pd in *picante*) which uses phylogenetic distance to compute within-sample diversity, and as measure of evenness, the Shannon diversity index, defined as $H=-\sum_{i=1}^s\frac{p_s\times \log \left({p}_s\right)}{\log (N)}$ with *s* ASV in a sample, *p* the proportion of reads of *s*, and *N* the total number of ASVs in the sample. Season and cumulative rainfall were highly collinear (*VIF* = 5.2), and we therefore chose to include only cumulative rainfall as it was shown to influence alpha-diversity in another primate species [40].

We used generalized linear mixed models (GLMMs) on alpha-diversity to test the influence of female’s age while controlling for cumulative rainfall, whether the female was gestating at the time of sampling (i.e., date of parturition minus average gestation length; detailed definition in supplementary material), and social group identity. Gestation (binary term with reference 0 indicating no gestation), and social group were included as fixed effects term, and the interaction age × cumulative rainfall was included as fixed effects term and as a random slope within the random intercept of individual’s identity (ID) to account for repeated sampling and keep type I error rate at 5% [98]. We excluded the correlation between random intercept and slope to allow models to converge. To account for differences in sequencing depth, we included the total number of reads per sample as a fixed term in the model on FPD, and the log transformed read counts as an offset term in the model on richness. We used a negative binomial error distribution and log link function for richness and a beta error distribution and logit link function for *H* (values bound between 0 and 1), fitted with the function glmmTMB from the eponym package. Significance of fixed effects terms was determined by likelihood ratio test using the function drop1 [99]. A Gaussian error distribution was fitted with the function lmer from the package *lmerTest* for FPD, which allows to assess significance of fixed effects terms by means of the Satterthwaite approximation [100] and a model fitted with restricted maximum likelihood. We determined model stability by dropping levels of random effects terms one at a time and compared the resulting model estimates to those derived from the full model, which revealed stability was no issue. Inspection of variance inflation factors (VIF) obtained with the function vif of the package *car* on corresponding linear models lacking the interaction and random terms did not reveal issues arising from collinearity (all *VIF* < 1.2). None of the assumptions on model error distribution was violated (normal distribution of residual in Gaussian models; lack of overdispersion in the negative binomial and beta models with overdispersion equal to 0.95 and 1.1, respectively). Full models were compared to their respective reference models comprising only control predictors (here cumulative rainfall, gestation, and group), the random slope and intercept structures, with number of reads as a fixed or offset term when applicable. Such full-reference model comparison avoids “cryptic multiple testing” [101]. Full-reference model comparisons were performed in the function ANOVA, with parameter test set to “Chisq.” We computed 95% confidence intervals of model estimates with parametric bootstrapping (*N* = 1000 bootstraps; function bootMer in *lme4*).

### Changes in bacterial taxa relative abundance and depletion of core microbiota with age

To determine whether changes in gut microbiota with age were associated with a depletion of the core (those taxa present in at least 90% of the samples [24]) in favor of a rise in opportunist and pathogen bacteria, we investigated changes in relative abundance with age at the phylum, family, and genus level. Read counts were summed from single ASVs counts at the desired level of analysis and transformed to relative abundance within sample. As a beta error distribution does not allow for values of exactly 0 or 1, we applied the transformation recommended by Smithson and Verkuilen [102] to shrink the response towards 0.5, which avoids adding arbitrary pseudo-counts: $f(x)=\frac{x\ast \left(n-1\right)+0.5}{n}$ with *n* = total number of data points in the sample and *x* the individual relative proportions.

We used a GLMM with beta error distribution (function glmmTMB in *glmmTMB*) to model the influence of age on a taxon’s relative abundance while controlling for season, gestation, and social group included as fixed effects terms. The model included the random slope of age and season within the random effects term ID. The beta distribution comprises a free dispersion parameter which usually is assumed to be constant across the entire dataset. However, this assumption is not always met, and, hence, we explicitly modeled the dispersion parameter as a function of age to overcome convergence problems. Sample’s read count was included as a weighting term. Significance was assessed with likelihood ratio tests against the reference model excluding age (function ANOVA with argument set to “Chisq”). When significant, *p*-values for the effect of age extracted from drop1 were corrected for overdispersion [103] and multiple testing (function p.adjust with p.adjust.methods = fdr). The models for six families and eleven genera did not converge and were excluded from the results. The dispersion parameter was left free to allow the model for the phylum *Actinobacteriota* to converge. Restricting the datasets to known genera (103 out of the 136 models that converged) did not modify the results regarding the repartition of core and noncore genera among those increasing, decreasing, or showing no change with age.

### Relationship between age, personal gut bacterial signature, and stability over time

To estimate between sample dissimilarity in bacterial composition (i.e., beta diversity), we rarefied sequencing depth at minimum read counts (9653 reads; Fig. S8). We estimated dissimilarity with three standard and complementary metrics: Bray-Curtis (BC) dissimilarity (function vegdist in *vegan*) assessing composition dissimilarity, weighted unifrac weighting composition with the phylogenetic relationship between taxa, and unweighted unifrac comparing composition based on presence/absence of taxa (respectively Wunifrac and UWunifrac hereafter; function GUnifrac in the eponym package) on the rarefied dataset.

To test whether gut microbiota composition carried a host personal signature, we tested the influence of ID on dissimilarity. We calculated with a Mantel test the Spearman correlation between the dissimilarity matrix (pairwise comparison between all samples) and a matrix indicating whether samples were collected from the same or different individuals. To then focus on the change of intraindividual dissimilarity over time, we restricted dissimilarity matrices to intraindividual pairwise comparisons and determined with a Mantel test the Spearman correlation between the intraindividual dissimilarity matrix and a second matrix indicating the time gap in days between collection of two samples. *P*-values for the Mantel tests were derived from 10,000 permutations which were restricted such that randomization took place only within individuals. To further investigate whether the change in microbiota composition over time could be associated with female’s age, we extracted for each female the correlation between intraindividual dissimilarity and time gap between sample collection. This gave one correlation coefficient per individual ranging between −1 and 1. This coefficient can be thought of as an estimate of the strength and direction of the evolution of intraindividual dissimilarity over time. The difference in intraindividual change of microbiota composition over time between age categories was assessed with a nonparametric Kruskal-Wallis test.

### Age as a predictor of gut bacterial composition

One underlying assumption in gut microbial research is that age could be associated with a specific composition across aging individuals. To assess the effect of age on dissimilarity in bacterial composition between samples, we ran permutational multivariate analyses of variance (PERMANOVA) with the function adonis in *vegan* (10,000 permutations) with first only ID and second season, gestation, group, and age on dissimilarity, with ID as blocking factor (“strata”) to control for repeated sampling.

### Personalization of gut bacterial communities and age

Then, to investigate whether individuals differed in the degree of personalization of their gut bacterial communities, we estimated the extent to which sample bacterial composition was unique to the individual. We restricted the dissimilarity matrix to pairwise comparisons between samples from different individuals from the same group and collected during the same season. For each sample, we kept only the lowest dissimilarity measured. This metric thus represents a measure of distance to “the closest neighbor,” in this case the sample collected from another individual in the same group and season with the least dissimilar composition [11]. We then fitted GLMMs with beta error distribution (function glmmTMB in *glmmTMB*) and logit link function to test the influence of age, season, gestation, and group on minimal dissimilarity based on either BC, Wunifrac, or UWunifrac. We also included the interaction between age and season and again modeled the dispersion of the beta distribution as a function of age after preliminary visualization of the relationship between age and minimal dissimilarity (confirmed by greater log likelihood of models including compared to excluding the dispersion argument). In essence, this effect estimates the extent to which the variability in the relative abundance depended on age. ID was included as a random effect term to control for repeated sampling, with random slopes of age and season within ID. We tested models’ stability by excluding levels of the random effect one at a time and comparing these estimates with those derived from the full dataset. Models did not show evidence of overdispersion (range = 0.88–0.97), and VIFs were all under 1.5 which indicate that collinearity was no issue. We assessed the significance of full models against their respective reference models lacking the interaction and fixed effects terms for age and season with likelihood ratio test (function ANOVA with argument set to “Chisq”) and extracted *p*-values for the fixed effects with the function drop1. The dispersion parameter was removed to allow drop1 to converge for Wunifrac which does not modify interpretation of the results. We computed 95% confidence intervals of model estimates using parametric bootstrapping (*N* = 1000 bootstraps; function simulate of the package *glmmTMB*).

### Progressive decreasing social activity with age as a possible driver of personalization

Sample size was 431 dyads for this analysis. Finally, we tested whether social interactions promote the transfer of gut microbiota between subjects and explain the age effects documented. To do so, frequency and duration of grooming interactions between pairs of females calculated over the entire study period (June 2018 to December 2019) were corrected for dyadic observation time, corresponding to the sum of observation time for each dyad member during which the other member of the dyad was also an adult and present in the group at the time. To test whether dyads interacting more frequently or for a longer time exhibited lower microbiota dissimilarity, we computed the average dissimilarity per dyad and used Bayesian regression multimembership models in *brms*. Such models are needed to account for the inherent dyadic nature of the data, which can be specified in the random term structure of the model [15, 104]. We fitted the model with beta error structure and logit-link function, including the fixed effects of dyadic grooming frequency or duration, controlling for group membership, a random effects (multimembership) term for the IDs of the individuals of a dyad, and a random slope of the frequency or duration. To penalize extreme estimates, we used regularizing priors β~normal (0, 1) for fixed effects terms, and the default priors student-t (3, 0.7, 0.25) for the random intercept, student-t (3, 0, 2.5) for the random slope terms, and R—lkj (1) for the correlation between the random intercept and slope. Evidence for a relationship between social interactions and microbiota dissimilarity is summarized by reporting estimates with estimated error, the lower and higher boundaries of the 90% credible interval (90CI), and the probability of a negative effect, i.e., the proportion of posterior samples smaller than 0 (*p*_−_). In this case, *p*_−_ can be interpreted as the probability in support of a social transmission of microbiota between individuals, with increasing values towards 1 providing greater support for the hypothesis. We ran 4000 iterations, with 2000 warmup on 4 chains, with adapt_delta set at 0.91, and a max_treedepth of 20. All chains converged as shown by inspections of caterpillar plots and Rhats equal to 1. Posterior sampling was sufficient for accurately estimating the effect of interactions on dissimilarity (both Bulk_ESS > 4000).

For the last analyses, sample size was 51 females. To further test whether microbiota personalization could be linked to the level of engagement in social interactions, we extracted the frequency and duration of grooming with a female’s closest partner, i.e., the adult female with whom she groomed most frequently or longest (top partner), and a female’s average grooming frequency and duration with all females in her social group. We fitted multiple regression models with the function lm [99] to analyze the relationship between female age and the frequency or duration of top partner and average grooming while correcting for social group. Model assumptions on residuals, absence of influential cases, and absence of multicollinearity were checked with residuals plotted against fitted values, DFBetas and VIFs. None of these indicated issues regarding model assumptions of stability or collinearity problems. Inspection of predictors effect sizes and *p*-values was used to assess significance [101].

## Supplementary Information

**Additional file 1: Supplementary file 1: Fig. S1**. Number of ASV observed per sample. **Fig. S2**. Number of samples in which a given ASV was observed. ASV present in only 1 sample across adult females were removed from the datasets. **Fig. S3**. Diversity did not vary with age in adult female macaques. Each data point indicates the diversity measured in one fecal sample. **Fig. S4**. (a) Prevalence of the bacterial genera exhibiting a negative, positive, or no change in relative abundance with age on an ASVs table non-filtered at 0.25% relative abundance. (b) Proportion of core and noncore taxa among the bacterial genera decreasing, showing no change, or increasing in relative abundance with age on an ASVs table non-filtered at 0.25% relative abundance. Results match the pattern found in the filtered ASVs dataset reported in the main text, specifically an increase in genera belonging to the noncore microbiome in the absence of major changes in the core. **Fig. S5**. Time gap (in days) between the collection of two samples from the same individual (a) in the full dataset or (c) between samples collected in different seasons. Average individual time elapsed between the collection of two samples was (b) 190 ± 71 days in the full dataset and (d) 287 ± 30 days in the dataset restricted to comparisons between seasons. The restricted dataset removed a large part of the variation between individuals, and removed samples collected only a few days apart. **Fig. S6**. (a) Technical replicates (sets 1-5) cluster together compared to all non-replicated samples (in grey), and (b) even more clearly when compared among each other. Dissimilarity between samples is summarized along the two axes of a nonmetric multidimensional scaling (NMDS). (c) Dissimilarity is lower between technical replicates than between samples. **Fig. S7**. Number of reads per sample. **Fig. S8**. Rarefaction curves. Analyses were run on the dataset at 9653 reads (red vertical line). **Table S3**. Relative abundance and prevalence of the 10 most abundant phyla. **Table S10**. Results of Bayesian multimembership models estimating the influence of dyadic interaction (A) frequency and (B) duration on microbiota composition dissimilarity. **Table S11**. Results of models with (A) grooming frequency to the closest social partner, (B) average frequency to all partners, (C) grooming duration to the closest social partner, (D) and average duration to all partners.

**Additional file 2: Supplementary file 2**: **Table S1-2**. Taxonomic characterization of the gut bacterial communities of adult female Assamese macaques. **Table S4**. Diversity of the gut bacterial community does not vary with age. **Table S5**. Relative abundance of rare bacterial taxa changes with age. **Table S6**. Taxonomic composition of the Assamese macaques females gut microbiota at the Phylum, Class, Order and Genus level. **Table S7-8**. Gut bacterial composition exhibits a personal signature and stability decreases with age. **Table S9**. Personalization of gut bacterial communities increases with age. **Table S12**. Progressive decreasing social activity with age as a possible driver of gut bacteria personalization. **Table S13-15**. Results based on the unfiltered dataset for spurious sequences (see methods section of the main text for details).

## Data Availability

The datasets generated during and/or analyzed during the current study, and the R codes used to generate analyses and figures, are available in the figshare repository with the identifier 10.6084/m9.figshare.19071182. The raw 16S RNA gene sequencing data are available in the BioProject database (ID: PRJNA795139) of the NCBI repository http://www.ncbi.nlm.nih.gov/bioproject/795139.

## References

[1] An R, Wilms E, Masclee AAM, Smidt H, Zoetendal EG, Jonkers D (2018). Age-dependent changes in GI physiology and microbiota: time to reconsider?. Gut BMJ Publishing Group.

[2] Badal VD, Vaccariello ED, Murray ER, Yu KE, Knight R, Jeste DV (2020). The gut microbiome, aging, and longevity: a systematic review. Nutrients.

[3] Bana B, Cabreiro F (2019). The microbiome and aging. Annu Rev Genet.

[4] Gupta VK, Kim M, Bakshi U, Cunningham KY, Davis JM, Lazaridis KN (2020). A predictive index for health status using species-level gut microbiome profiling. Nat Commun. Nat Commun Nature Publishing Group.

[5] Agirman G, Yu KB, Hsiao EY (2021). Signaling inflammation across the gut-brain axis. Science Am Assoc Advancement Sci.

[6] Belkaid Y, Hand TW (2014). Role of the microbiota in immunity and inflammation. Cell..

[7] Cammarota G, Ianiro G, Gasbarrini A (2014). Fecal microbiota transplantation for the treatment of Clostridium difficile infection: a systematic review. J Clin Gastroenterol.

[8] Clayton JB, Gomez A, Amato K, Knights D, Travis DA, Blekhman R (2018). The gut microbiome of nonhuman primates: lessons in ecology and evolution. Am J Primatol.

[9] Ghosh TS, Das M, Jeffery IB, O’Toole PW. In: Turnbaugh P, Garrett WS, Lozupone CA, Turnbaugh P, editors. Adjusting for age improves identification of gut microbiome alterations in multiple diseases. eLife. eLife Sciences Publications, Ltd. 2020;9:e50240. 10.7554/eLife.50240 PMC7065848 32159510

[10] Smith P, Willemsen D, Popkes M, Metge F, Gandiwa E, Reichard M, et al. Regulation of life span by the gut microbiota in the short-lived African turquoise killifish. Dillin A. eLife. eLife Sciences Publications, Ltd. 2017;6:e27014. 10.7554/eLife.27014 PMC5566455 28826469

[11] Wilmanski T, Diener C, Rappaport N, Patwardhan S, Wiedrick J, Lapidus J (2021). Gut microbiome pattern reflects healthy ageing and predicts survival in humans. Nat Metab Nature Publishing Group.

[12] Wilson QN, Wells M, Davis AT, Sherrill C, Tsilimigras MCB, Jones RB (2018). Greater microbial translocation and vulnerability to metabolic disease in healthy aged female monkeys. Sci Rep.

[13] Galbany J, Altmann J, Pérez-Pérez A, Alberts SC (2011). Age and individual foraging behavior predict tooth wear in Amboseli baboons. Am J Phys Anthropol.

[14] Charles ST, Carstensen LL (2010). Social and emotional aging. Annu Rev Psychol.

[15] Raulo A, Allen BE, Troitsky T, Husby A, Firth JA, Coulson T (2021). Social networks strongly predict the gut microbiota of wild mice. ISME J.

[16] Raulo A, Ruokolainen L, Lane A, Amato K, Knight R, Leigh S (2018). Social behaviour and gut microbiota in red-bellied lemurs (*Eulemur rubriventer*): in search of the role of immunity in the evolution of sociality. J Anim Ecol.

[17] Sarkar A, Harty S, Johnson KV-A, Moeller AH, Archie EA, Schell LD (2020). Microbial transmission in animal social networks and the social microbiome. Nat Ecol Evol Nature Publishing Group.

[18] López-Otín C, Blasco MA, Partridge L, Serrano M, Kroemer G (2013). The hallmarks of aging. Cell..

[19] van den Beld AW, Kaufman J-M, Zillikens MC, Lamberts SWJ, Egan JM, van der Lely AJ (2018). The physiology of endocrine systems with ageing. Lancet Diabetes Endocrinol.

[20] Dansereau G, Wey TW, Legault V, Brunet MA, Kemnitz JW, Ferrucci L (2019). Conservation of physiological dysregulation signatures of aging across primates. Aging Cell.

[21] Fulop T, Dupuis G, Baehl S, Le Page A, Bourgade K, Frost E (2016). From inflamm-aging to immune-paralysis: a slippery slope during aging for immune-adaptation. Biogerontology..

[22] Biagi E, Franceschi C, Rampelli S, Severgnini M, Ostan R, Turroni S (2016). Gut microbiota and extreme longevity. Curr Biol.

[23] O’Toole PW, Jeffery IB (2015). Gut microbiota and aging. Science..

[24] Risely A (2020). Applying the core microbiome to understand host–microbe systems. J Anim Ecol.

[25] Popkes M, Valenzano DR. Microbiota–host interactions shape ageing dynamics. Philos Trans R Soc B Biol Sci Royal Society; 2020;375:20190596. 10.1098/rstb.2019.0596 PMC7435156 32772667

[26] Ragonnaud E, Biragyn A (2021). Gut microbiota as the key controllers of “healthy” aging of elderly people. Immun Ageing.

[27] Frost F, Kacprowski T, Rühlemann M, Pietzner M, Bang C, Franke A (2021). Long-term instability of the intestinal microbiome is associated with metabolic liver disease, low microbiota diversity, diabetes mellitus and impaired exocrine pancreatic function. Gut BMJ Publishing Group.

[28] Tap J, Furet J-P, Bensaada M, Philippe C, Roth H, Rabot S (2015). Gut microbiota richness promotes its stability upon increased dietary fibre intake in healthy adults. Environ Microbiol.

[29] Leite G, Pimentel M, Barlow GM, Chang C, Hosseini A, Wang J (2021). Age and the aging process significantly alter the small bowel microbiome. Cell Rep.

[30] Maffei VJ, Kim S, Blanchard E, Luo M, Jazwinski SM, Taylor CM (2017). Biological aging and the human gut microbiota. J Gerontol Ser A.

[31] Claesson MJ, Jeffery IB, Conde S, Power SE, O’Connor EM, Cusack S (2012). Gut microbiota composition correlates with diet and health in the elderly. Nature. Nature Publishing Group.

[32] Claesson MJ, Cusack S, O’Sullivan O, Greene-Diniz R, de WH, Flannery E (2011). Composition, variability, and temporal stability of the intestinal microbiota of the elderly. Proc Natl Acad Sci.

[33] Ale EC, Binetti AG (2021). Role of probiotics, prebiotics, and synbiotics in the elderly: insights into their applications. Front Microbiol.

[34] Grieneisen L, Dasari M, Gould TJ, Björk JR, Grenier J-C, Yotova V (2021). Gut microbiome heritability is nearly universal but environmentally contingent. Science..

[35] Moeller AH, Foerster S, Wilson ML, Pusey AE, Hahn BH, Ochman H (2016). Social behavior shapes the chimpanzee pan-microbiome. Sci Adv.

[36] Perofsky AC, Lewis RJ, Abondano LA, Di Fiore A, Meyers LA (2017). Hierarchical social networks shape gut microbial composition in wild Verreaux’s sifaka. Proc R Soc B Biol Sci Royal Society.

[37] Thevaranjan N, Puchta A, Schulz C, Naidoo A, Szamosi JC, Verschoor CP (2017). Age-associated microbial dysbiosis promotes intestinal permeability, systemic inflammation, and macrophage dysfunction. Cell Host Microbe.

[38] Bowerman KL, Knowles SCL, Bradley JE, Baltrūnaitė L, Lynch MDJ, Jones KM (2021). Effects of laboratory domestication on the rodent gut microbiome. ISME Commun.

[39] Narat V, Amato KR, Ranger N, Salmona M, Mercier-Delarue S, Rupp S (2020). A multi-disciplinary comparison of great ape gut microbiota in a Central African forest and European zoo. Sci Rep.

[40] Baniel A, Amato KR, Beehner JC, Bergman TJ, Mercer A, Perlman RF (2021). Seasonal shifts in the gut microbiome indicate plastic responses to diet in wild geladas. Microbiome..

[41] Risely A, Wilhelm K, Clutton-Brock T, Manser MB, Sommer S (2021). Diurnal oscillations in gut bacterial load and composition eclipse seasonal and lifetime dynamics in wild meerkats. Nat Commun.

[42] Janiak MC, Montague MJ, Villamil CI, Stock MK, Trujillo AE, DePasquale AN (2021). Age and sex-associated variation in the multi-site microbiome of an entire social group of free-ranging rhesus macaques. Microbiome..

[43] Perofsky AC, Ancel Meyers L, Abondano LA, Di Fiore A, Lewis RJ (2021). Social groups constrain the spatiotemporal dynamics of wild sifaka gut microbiomes. Mol Ecol.

[44] Reese AT, Phillips SR, Owens LA, Venable EM, Langergraber KE, Machanda ZP (2021). Age patterning in wild chimpanzee gut microbiota diversity reveals differences from humans in early life. Curr Biol.

[45] Trosvik P, de Muinck EJ, Rueness EK, Fashing PJ, Beierschmitt EC, Callingham KR (2018). Multilevel social structure and diet shape the gut microbiota of the gelada monkey, the only grazing primate. Microbiome..

[46] Qiu X, Zhao X, Cui X, Mao X, Tang N, Jiao C (2020). Characterization of fungal and bacterial dysbiosis in young adult Chinese patients with Crohn’s disease. Ther Adv Gastroenterol. SAGE Publications Ltd STM.

[47] Takeshita K, Mizuno S, Mikami Y, Sujino T, Saigusa K, Matsuoka K (2016). A single species of Clostridium subcluster XIVa decreased in ulcerative colitis patients. Inflamm Bowel Dis.

[48] Tamanai-Shacoori Z, Smida I, Bousarghin L, Loreal O, Meuric V, Fong SB (2017). Roseburia spp.: a marker of health?. Future Microbiol.

[49] Romaní-Pérez M, López-Almela I, Bullich-Vilarrubias C, Rueda-Ruzafa L, Gómez Del Pulgar EM, Benítez-Páez A (2021). Holdemanella biformis improves glucose tolerance and regulates GLP-1 signaling in obese mice. FASEB J.

[50] Pujo J, Petitfils C, Faouder PL, Eeckhaut V, Payros G, Maurel S (2021). Bacteria-derived long chain fatty acid exhibits anti-inflammatory properties in colitis. Gut BMJ Publishing Group.

[51] Parker BJ, Wearsch PA, Veloo ACM, Rodriguez-Palacios A (2020). The genus Alistipes: gut bacteria with emerging implications to inflammation, cancer, and mental health. Front Immunol.

[52] Amato KR, Metcalf JL, Song SJ, Hale VL, Clayton J, Ackermann G (2016). Using the gut microbiota as a novel tool for examining colobine primate GI health. Glob Ecol Conserv.

[53] Faith JJ, Guruge JL, Charbonneau M, Subramanian S, Seedorf H, Goodman AL, et al. The long-term stability of the human gut microbiota. Science Am Assoc Advancement Sci; 2013;341:1237439. 10.1126/science.1237439 PMC3791589 23828941

[54] Johnson AJ, Vangay P, Al-Ghalith GA, Hillmann BM, Ward TL, Shields-Cutler RR (2019). Daily sampling reveals personalized diet-microbiome associations in humans. Cell Host Microbe.

[55] Lloyd-Price J, Mahurkar A, Rahnavard G, Crabtree J, Orvis J, Hall AB (2017). Strains, functions and dynamics in the expanded Human Microbiome Project. Nature. Nature Publishing Group.

[56] Schloissnig S, Arumugam M, Sunagawa S, Mitreva M, Tap J, Zhu A (2013). Genomic variation landscape of the human gut microbiome. Nature. Nature Publishing Group.

[57] Zmora N, Zeevi D, Korem T, Segal E, Elinav E (2016). Taking it personally: personalized utilization of the human microbiome in health and disease. Cell Host Microbe.

[58] Flores GE, Caporaso JG, Henley JB, Rideout JR, Domogala D, Chase J (2014). Temporal variability is a personalized feature of the human microbiome. Genome Biol.

[59] Ren T, Grieneisen LE, Alberts SC, Archie EA, Wu M (2016). Development, diet, and dynamism: longitudinal and cross-sectional predictors of gut microbial communities in wild baboons. Environ Microbiol.

[60] Suvorov A, Karaseva A, Kotyleva M, Kondratenko Y, Lavrenova N, Korobeynikov A (2018). Autoprobiotics as an approach for restoration of personalised microbiota. Front Microbiol.

[61] Hooks KB, O’Malley MA (2017). Dysbiosis and its discontents mBio.

[62] Louca S, Polz MF, Mazel F, Albright MBN, Huber JA, O’Connor MI (2018). Function and functional redundancy in microbial systems. Nat Ecol Evol Nature Publishing Group.

[63] Jeffery IB, Lynch DB, O’Toole PW (2016). Composition and temporal stability of the gut microbiota in older persons. ISME J Nature Publishing Group.

[64] Degnan PH, Pusey AE, Lonsdorf EV, Goodall J, Wroblewski EE, Wilson ML (2012). Factors associated with the diversification of the gut microbial communities within chimpanzees from Gombe National Park. Proc Natl Acad Sci.

[65] Sato Y, Atarashi K, Plichta DR, Arai Y, Sasajima S, Kearney SM (2021). Novel bile acid biosynthetic pathways are enriched in the microbiome of centenarians. Nature. Nature Publishing Group.

[66] Wang J, Chen W-D, Wang Y-D (2020). The relationship between gut microbiota and inflammatory diseases: the role of macrophages. Front Microbiol.

[67] Wei Z-Y, Rao J-H, Tang M-T, Zhao G-A, Li Q-C, Wu L-M, et al. Characterization of changes and driver microbes in gut microbiota during healthy aging using a captive monkey model. Genomics Proteomics Bioinformatics. 2021. 10.1016/j.gpb.2021.09.009. 10.1016/j.gpb.2021.09.009 PMC9684162 34974191

[68] Chiou KL, Montague MJ, Goldman EA, Watowich MM, Sams SN, Song J (2020). Rhesus macaques as a tractable physiological model of human ageing. Philos Trans R Soc B Biol Sci Royal Society.

[69] Adriansjach J, Baum ST, Lefkowitz EJ, Van Der Pol WJ, Buford TW, Colman RJ (2020). Age-related differences in the gut microbiome of rhesus macaques. J Gerontol Ser A.

[70] Duan J, Yin B, Li W, Chai T, Liang W, Huang Y (2019). Age-related changes in microbial composition and function in cynomolgus macaques. Aging..

[71] Bennett G, Malone M, Sauther ML, Cuozzo FP, White B, Nelson KE (2016). Host age, social group, and habitat type influence the gut microbiota of wild ring-tailed lemurs (Lemur catta). Am J Primatol.

[72] Machanda ZP, Rosati AG. Shifting sociality during primate ageing. Philos Trans R Soc B Biol Sci Royal Society; 2020;375:20190620. 10.1098/rstb.2019.0620 PMC7540961 32951557

[73] Grieneisen LE, Livermore J, Alberts S, Tung J, Archie EA (2017). Group living and male dispersal predict the core gut microbiome in wild baboons. Integr Comp Biol Oxford Academic.

[74] Petrullo L, Ren T, Wu M, Boonstra R, Palme R, Boutin S (2022). Glucocorticoids coordinate changes in gut microbiome composition in wild North American red squirrels. Sci Rep Nature Publishing Group.

[75] Uren Webster TM, Rodriguez-Barreto D, Consuegra S, Garcia de Leaniz C. Cortisol-related signatures of stress in the fish microbiome. Front Microbiol. 2020;11:1621. 10.3389/fmicb.2020.01621 PMC7381252 32765459

[76] Lee DS, Kang YHR, Ruiz-Lambides AV, Higham JP (2021). The observed pattern and hidden process of female reproductive trajectories across the life span in a non-human primate. J Anim Ecol.

[77] Colman RJ, McKiernan SH, Aiken JM, Weindruch R (2005). Muscle mass loss in rhesus monkeys: age of onset. Exp Gerontol.

[78] Simmons HA (2016). Age-associated pathology in rhesus macaques (Macaca mulatta). Vet Pathol SAGE Publications Inc.

[79] Cerroni AM, Tomlinson GA, Turnquist JE, Grynpas MD (2000). Bone mineral density, osteopenia, and osteoporosis in the rhesus macaques of Cayo Santiago. Am J Phys Anthropol.

[80] Worsley SF, Davies CS, Mannarelli M-E, Hutchings MI, Komdeur J, Burke T (2021). Gut microbiome composition, not alpha diversity, is associated with survival in a natural vertebrate population. Anim Microbiome.

[81] Borries C, Lu A, Ossi-Lupo K, Larney E, Koenig A (2011). Primate life histories and dietary adaptations: a comparison of asian colobines and macaques. Am J Phys Anthropol.

[82] Richter C, Heesen M, Nenadić O, Ostner J, Schülke O (2016). Males matter: increased home range size is associated with the number of resident males after controlling for ecological factors in wild Assamese macaques. Am J Phys Anthropol.

[83] Uno H (1997). Age-related pathology and biosenescent markers in captive rhesus macaques. AGE..

[84] Altmann J (1974). Observational study of behavior: sampling methods. Behaviour..

[85] Savtchenko A, Greenbelt M. Goddard Earth Sciences Data and Information Services Center TRMM (TMPA-RT) near real-time precipitation L3 1 day 0.25 degree x 0.25 degree V7; 2016. 10.5067/TRMM/TMPA/DAY-E/7. Accessed 1 June 2021.

[86] Klindworth A, Pruesse E, Schweer T, Peplies J, Quast C, Horn M (2013). Evaluation of general 16S ribosomal RNA gene PCR primers for classical and next-generation sequencing-based diversity studies. Nucleic Acids Res.

[87] Chen S, Zhou Y, Chen Y, Gu J (2018). fastp: an ultra-fast all-in-one FASTQ preprocessor. Bioinformatics..

[88] Zhang J, Kobert K, Flouri T, Stamatakis A (2014). PEAR: a fast and accurate Illumina paired-end reAd mergeR. Bioinformatics..

[89] Martin M (2011). Cutadapt removes adapter sequences from high-throughput sequencing reads. EMBnet.journal..

[90] Rognes T, Flouri T, Nichols B, Quince C, Mahé F (2016). VSEARCH: a versatile open source tool for metagenomics. PeerJ. PeerJ Inc.

[91] Edgar RC. UNOISE2: improved error-correction for Illumina 16S and ITS amplicon sequencing. bioRxiv. 2016:081257. 10.1101/081257.

[92] Quast C, Pruesse E, Yilmaz P, Gerken J, Schweer T, Yarza P (2013). The SILVA ribosomal RNA gene database project: improved data processing and web-based tools. Nucleic Acids Res.

[93] Gao X, Lin H, Revanna K, Dong Q (2017). A Bayesian taxonomic classification method for 16S rRNA gene sequences with improved species-level accuracy. BMC Bioinformatics.

[94] Reitmeier S, Hitch TCA, Treichel N, Fikas N, Hausmann B, Ramer-Tait AE (2021). Handling of spurious sequences affects the outcome of high-throughput 16S rRNA gene amplicon profiling. ISME Commun.

[95] Nearing JT, Douglas GM, Comeau AM, Langille MGI (2018). Denoising the denoisers: an independent evaluation of microbiome sequence error-correction approaches. PeerJ. PeerJ Inc.

[96] Katoh K, Standley DM (2013). MAFFT multiple sequence alignment software version 7: improvements in performance and usability. Mol Biol Evol.

[97] Price MN, Dehal PS, Arkin AP (2010). FastTree 2 – approximately maximum-likelihood trees for large alignments.. Plos One.

[98] Barr DJ, Levy R, Scheepers C, Tily HJ (2013). Random effects structure for confirmatory hypothesis testing: keep it maximal. J Mem Lang.

[99] R Core Team. R. A language and environment for statistical computing. R Foundation for statistical computing. Vienna, Austria; 2020. Available from: https://www.R-project.org/

[100] Luke SG (2017). Evaluating significance in linear mixed-effects models in R. Behav Res Methods.

[101] Forstmeier W, Schielzeth H (2011). Cryptic multiple hypotheses testing in linear models: overestimated effect sizes and the winner’s curse. Behav Ecol Sociobiol.

[102] Smithson M, Verkuilen J (2006). A better lemon squeezer? Maximum-likelihood regression with beta-distributed dependent variables. Psychol Methods.

[103] Gelman A, Hill J (2007). Data analysis using regression and multilevel/hierarchical models.

[104] Martin JS, Koski SE, Bugnyar T, Jaeggi AV, Massen JJM (2021). Prosociality, social tolerance and partner choice facilitate mutually beneficial cooperation in common marmosets, Callithrix jacchus. Anim Behav.

