# Supplementary material for: Aging gut microbiota of wild macaques are equally diverse, less stable, but progressively personalized
Source: Microbiome. 2022 Jun 19;10:95. doi: 10.1186/s40168-022-01283-2 (PMC9206754; doi:10.1186/s40168-022-01283-2)
Supplement: Supplementary file 2 — Additional file 1: Supplementary file 1: Fig. S1. Number of ASV observed per sample. Fig. S2. Number of samples in which a given ASV was observed. ASV present in only 1 sample across adult females were removed from the datasets. Fig. S3. Diversity did not vary with age in adult female macaques. Each data point indicates the diversity measured in one fecal sample. Fig. S4. (a) Prevalence of the bacterial genera exhibiting a negative, positive, or no change in relative abundance with age on an ASVs table non-filtered at 0.25% relative abundance. (b) Proportion of core and noncore taxa among the bacterial genera decreasing, showing no change, or increasing in relative abundance with age on an ASVs table non-filtered at 0.25% relative abundance. Results match the pattern found in the filtered ASVs dataset reported in the main text, specifically an increase in genera belonging to the noncore microbiome in the absence of major changes in the core. Fig. S5. Time gap (in days) between the collection of two samples from the same individual (a) in the full dataset or (c) between samples collected in different seasons. Average individual time elapsed between the collection of two samples was (b) 190 ± 71 days in the full dataset and (d) 287 ± 30 days in the dataset restricted to comparisons between seasons. The restricted dataset removed a large part of the variation between individuals, and removed samples collected only a few days apart. Fig. S6. (a) Technical replicates (sets 1-5) cluster together compared to all non-replicated samples (in grey), and (b) even more clearly when compared among each other. Dissimilarity between samples is summarized along the two axes of a nonmetric multidimensional scaling (NMDS). (c) Dissimilarity is lower between technical replicates than between samples. Fig. S7. Number of reads per sample. Fig. S8. Rarefaction curves. Analyses were run on the dataset at 9653 reads (red vertical line). Table S3. Relative abundance and prevalence of the 10 [file 40168_2022_1283_MOESM2_ESM.pdf]

Supplementary file 1 for

The aging gut microbiota of a wild living primate: equally diverse, less stable but progressively personalized

Baptiste Sadoughi, Dominik Schneider, Rolf Daniel, Oliver Schülke, Julia Ostner

Contact: Baptiste Sadoughi, bsadoug@uni-goettingen.de

Taxonomic characterization of the gut bacterial communities of adult female Assamese macaques

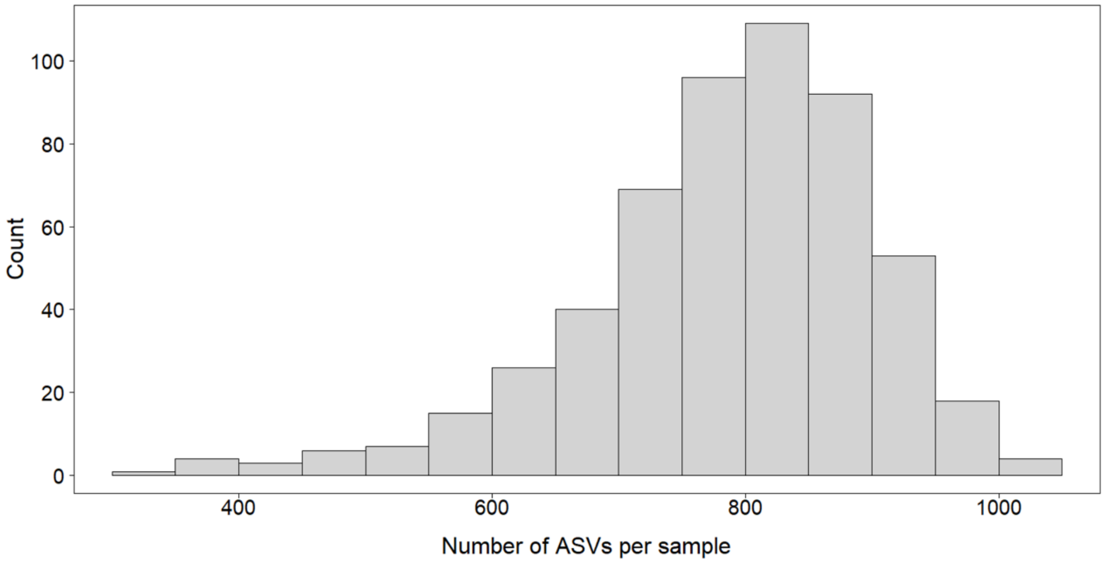

Figure S1. Number of ASV observed per sample.

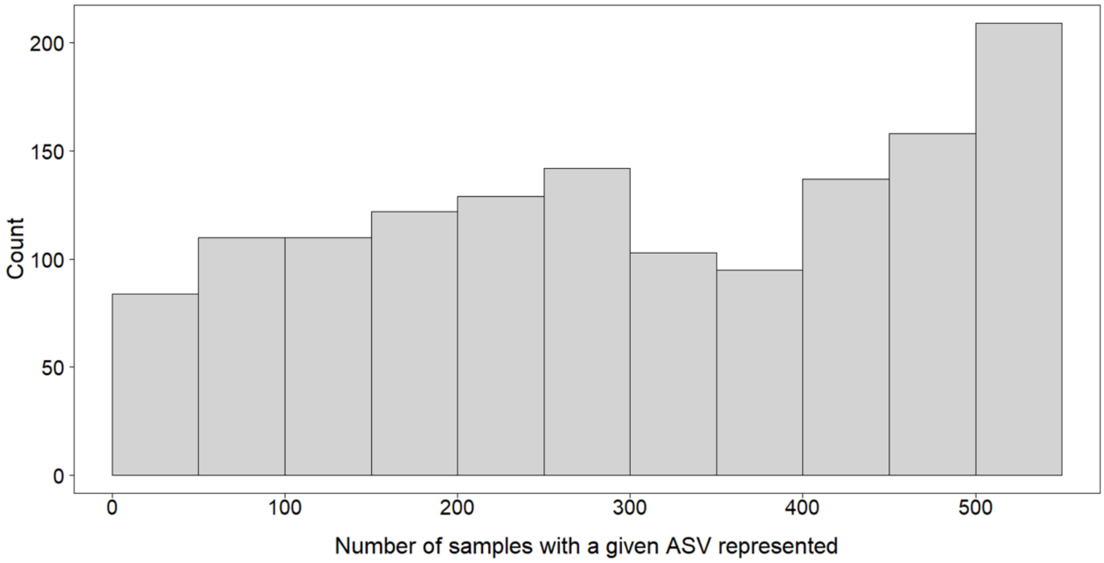

Figure S2. Number of samples in which a given ASV was observed. ASV present in only 1 sample across adult females were removed from the datasets.

Table S3. Relative abundance and prevalence of the 10 most abundant phyla.

Phylum	Mean Abundance (%)	Prevalence (%)
**Firmicutes**	56.93	100
**Bacteroidota**	17.19	100
**Spirochaetota**	11.06	100
**Proteobacteria**	4.43	100
**Verrucomicrobiota**	2.92	98.90
**Actinobacteriota**	2.74	100
**Cyanobacteria**	1.76	99.63
**Unclassified**	1.70	98.34
**Fibrobacterota**	0.47	90.61
**Desulfobacterota**	0.33	90.42

Diversity of the gut bacterial community does not vary with age

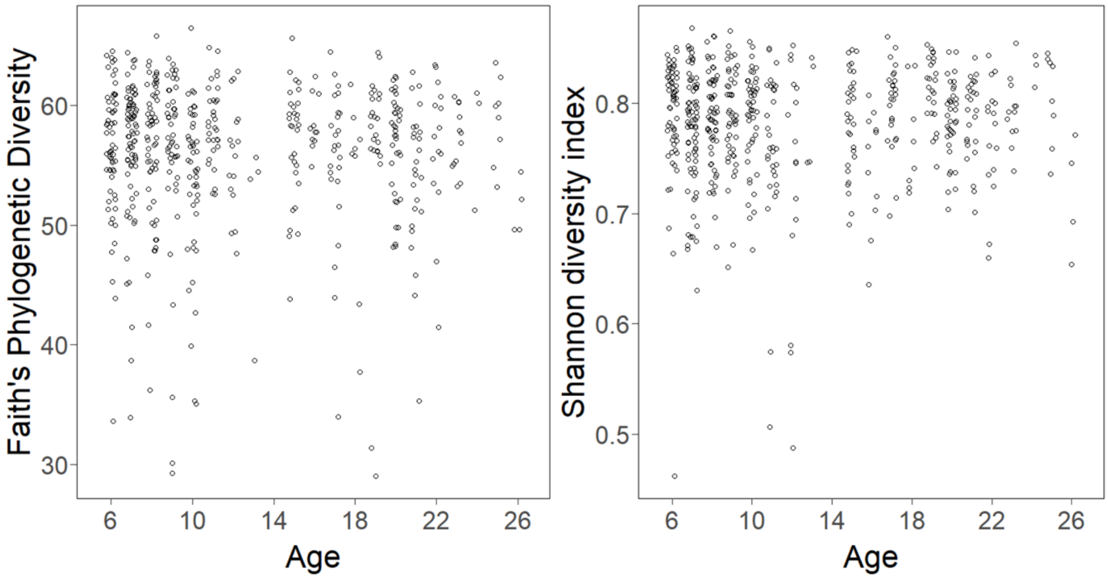

Figure S3. Diversity did not vary with age in adult female macaques. Each data point indicates the diversity measured in one fecal sample.

Relative abundance of rare bacterial taxa changes with age

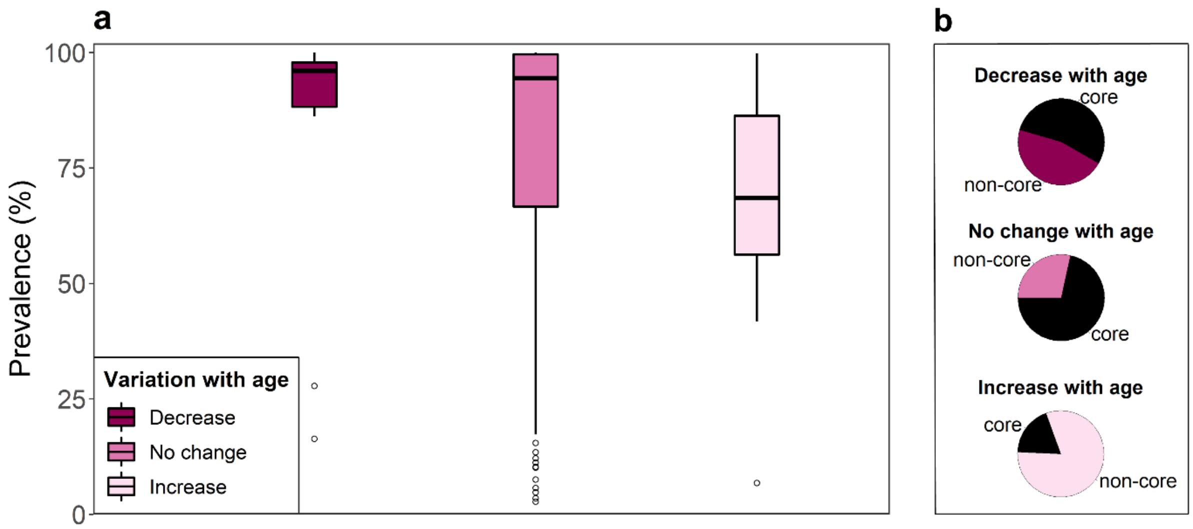

Figure S4. **a** Prevalence of the bacterial genera exhibiting a negative, positive, or no change in relative abundance with age on an ASVs table non-filtered at 0.25% relative abundance. **b** Proportion of core and noncore taxa among the bacterial genera decreasing, showing no change, or increasing in relative abundance with age on an ASVs table non-filtered at 0.25% relative abundance. Results match the pattern found in the filtered ASVs dataset reported in the main text, specifically an increase in genera belonging to the noncore microbiome in the absence of major changes in the core.

Gut bacterial composition exhibits a personal signature and stability decreases with age

Relationship between gut microbiota diversity and stability

The decrease in the stability of the gut bacterial composition in older females despite no variation in composition diversity with age appears in contradiction with several clinical studies showing that diversity at one time is associated with stability to a subsequent period [27,28,59,63]. To rule out that lower diversity was the underlying cause for reduced stability in the older females, we investigated the relationship between these two parameters. We assessed the correlation between diversity and composition stability, i.e., mean intraindividual dissimilarity across two seasons, to test whether a more diverse composition was associated with the stability of the gut bacterial composition. As the time gap between samples collected from the same individual varied greatly between females, which could bias our analysis, we restricted our dataset to intraindividual dissimilarities calculated between samples collected in different seasons, which successfully homogenized the time gap between females (Fig. S5). This left for analysis 45 adult females that were sampled across at least 2 seasons. The relationship between average individual alpha-diversity (three measures) against average intraindividual dissimilarity (also three measures) between seasons was assessed with simple

Spearman correlation and FDR-adjusted p-values (hereafter FDR-p). The order of seasons in time (1st rich, 2nd lean, 3rd intermediate) further gave the opportunity to investigate whether higher diversity at a starting point predicted greater stability. We calculated both predictive (i.e., initial diversity correlated with stability) and backward (i.e., final diversity correlated with stability) relationships to weight evidence for a causal influence of diversity on stability. Sample sizes were 45 paired diversity-stability metrics from 45 females.

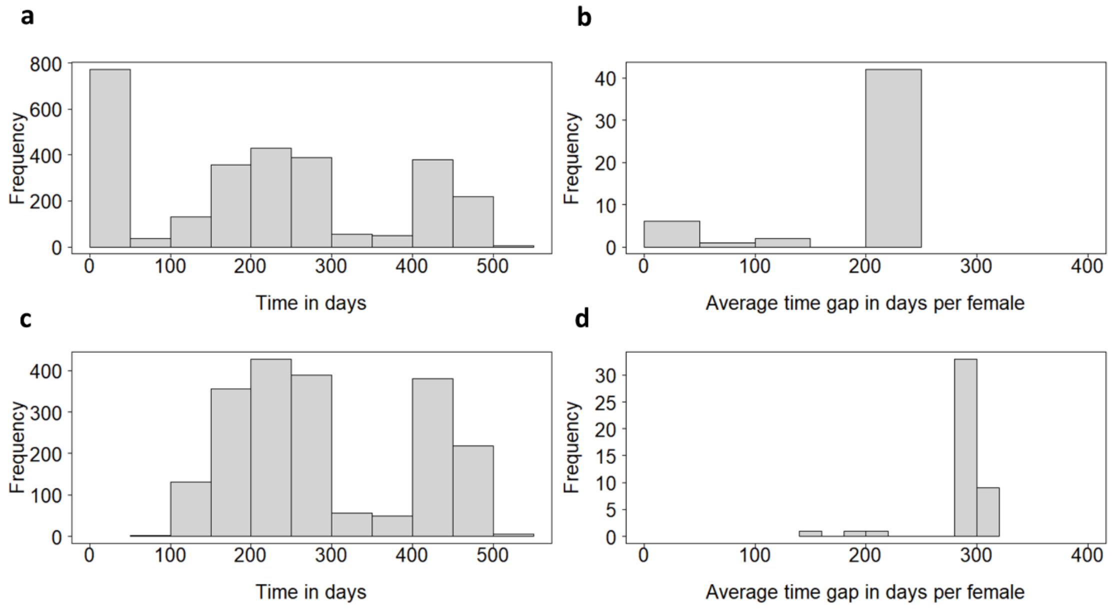

Figure S5. Time gap (in days) between the collection of two samples from the same individual **a** in the full dataset or **c** between samples collected in different seasons. Average individual time elapsed between the collection of two samples was **b** 190 ± 71 days in the full dataset and **d** 287 ± 30 days in the dataset restricted to comparisons between seasons. The restricted dataset removed a large part of the variation between individuals, and removed samples collected only a few days apart.

Results show that stability is not predicted by diversity of gut bacterial composition. In details, predictive pairwise correlations showed that diversity in the 1st season was associated with stability between the 1st-2nd and the 1st-3rd ($-0.87 \leq r \leq -0.44$, all FDR-p ≤ 0.001; Tab. S8), whereas diversity in the 2nd season was not associated with stability between the 2nd-3rd seasons ($-0.35 \leq r \leq 0.24$, 0.2 ≤ FDR-p ≤ 1). The backward correlations showed that diversity in the 2nd season was not associated with the stability between the 1st and 2nd seasons ($-0.31 \leq r \leq -0.21$, 0.3 ≤ FDR-p ≤ 0.4; Tab. S8), whereas the diversity in the 3rd season was associated with the stability between the 1st and 3rd and with that between the 2nd and 3rd season ($-0.88 \leq r \leq -0.53$, all FDR-p ≤ 0.001; Tab. S8). Therefore, despite some evidence for a link between diversity and stability, we cannot conclude that high initial diversity promotes long-term stability.

Progressive decreasing social activity with age as a possible driver of gut bacteria personalization

Table S10. Results of Bayesian multimembership models estimating the influence of dyadic interaction **A** frequency and **B** duration on microbiota composition dissimilarity.

	**Predictor**	**Estimate**	**Est.Error**	**$90CI_{lower}$**	**$90CI_{upper}$**
**A**	Intercept	0.59	0.06	0.48	0.71
	Dyadic interaction	-0.01	0.00	-0.02	0.00
	Group MST (ref MOT)	0.06	0.07	-0.08	0.20
	Group SST (ref MOT)	0.00	0.08	-0.16	0.17
**B**	Intercept	0.59	0.06	0.48	0.71
	Age	-0.01	0.00	-0.01	0.00
	Group MST (ref MOT)	0.06	0.07	-0.08	0.20
	Group SST (ref MOT)	0.00	0.08	-0.16	0.16

Table S11. Results of models with **A** grooming frequency to the closest social partner, **B** average frequency to all partners, **C** grooming duration to the closest social partner, **D** and average duration to all partners.

	Predictor	Estimate	SE	CI_{lower}	CI_{upper}	t	p
**A**	Intercept	-0.17	0.23	-0.63	0.29	-0.73	-
	**Age**	**-0.27**	**0.13**	**-0.52**	**-0.01**	**-2.12**	**0.04**
	Group MST (ref MOT)	-0.13	0.29	-0.73	0.46	-0.45	0.7
	**Group SST (ref MOT)**	**0.89**	**0.34**	**0.22**	**1.57**	**2.65**	**0.01**
**B**	Intercept	0.16	0.20	-0.25	0.57	0.80	-
	**Age**	**-0.27**	**0.11**	**-0.50**	**-0.05**	**-2.43**	**0.02**
	**Group MST (ref MOT)**	**-0.75**	**0.26**	**-1.28**	**-0.22**	**-2.87**	**0.006**
	**Group SST (ref MOT)**	**0.69**	**0.30**	**0.09**	**1.29**	**2.32**	**0.02**
**C**	Intercept	-0.30	0.23	-0.77	0.17	-1.30	-
	Age	-0.21	0.13	-0.47	0.04	-1.66	0.1
	Group MST (ref MOT)	0.08	0.30	-0.52	0.69	0.28	0.8
	**Group SST (ref MOT)**	**1.03**	**0.34**	**0.35**	**1.72**	**3.02**	**0.004**
**D**	Intercept	0.05	0.21	-0.37	0.47	0.22	0.8
	**Age**	**-0.23**	**0.11**	**-0.46**	**0.00**	**-2.02**	**0.05**
	**Group MST (ref MOT)**	**-0.58**	**0.27**	**-1.12**	**-0.04**	**-2.17**	**0.04**
	**Group SST (ref MOT)**	**0.85**	**0.31**	**0.23**	**1.46**	**2.76**	**0.008**

All full-null model comparisons were significant $p < 0.01$.

Methods

Fecal sample collection

Fruit phenology score

Fruit abundance was measured at the study site from tree species abundance in 45 botanical plots covering 21ha and monthly phenology scores from more than 650 trees representing important fruit sources (*Heesen et al., 2013 Behav Ecol Sociobiol*). We derived daily scores from those monthly scores, which we then multiplied by the number of samples collected on that day. Samples analyzed were collected during a rich season with high fruit availability (July through October 2018; daily fruit score mean ± SD = 43.3 ± 10.7, range: 28.5–59.5), a lean season (January through April 2019; daily fruit score

mean ± SD = 12.9 ± 7.3, range: 4.3–40.9) corresponding to low food availability, and a third intermediate season (October through December 2019; daily fruit score 14.2 ± 10.9, range: 5.5–50.4).

DNA extraction, amplification of 16S rRNA genes and sequencing

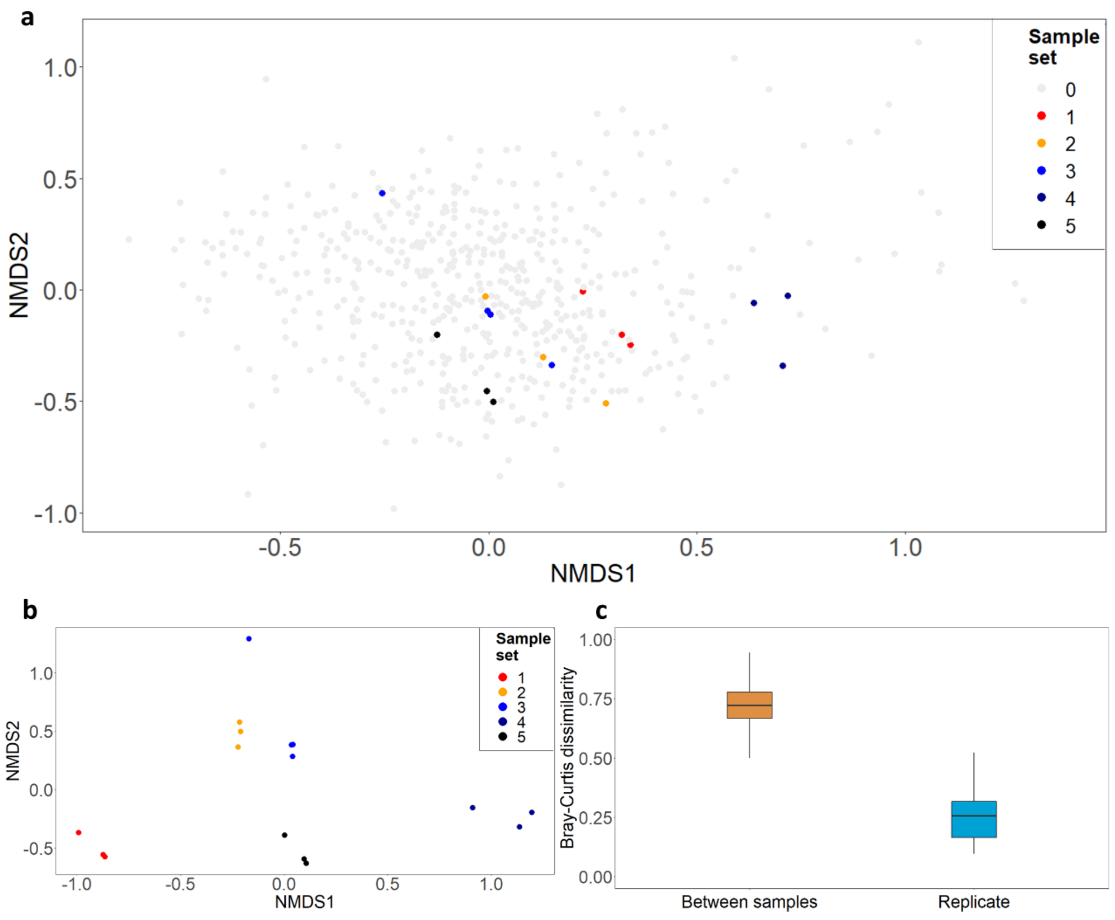

Figure S6. **a** Technical replicates (sets 1-5) cluster together compared to all non-replicated samples (in grey), and **b** even more clearly when compared among each other. Dissimilarity between samples is summarized along the two axes of a nonmetric multidimensional scaling (NMDS). **c** Dissimilarity is lower between technical replicates than between samples.

Sequence processing, taxonomic assignment, and dataset preparation

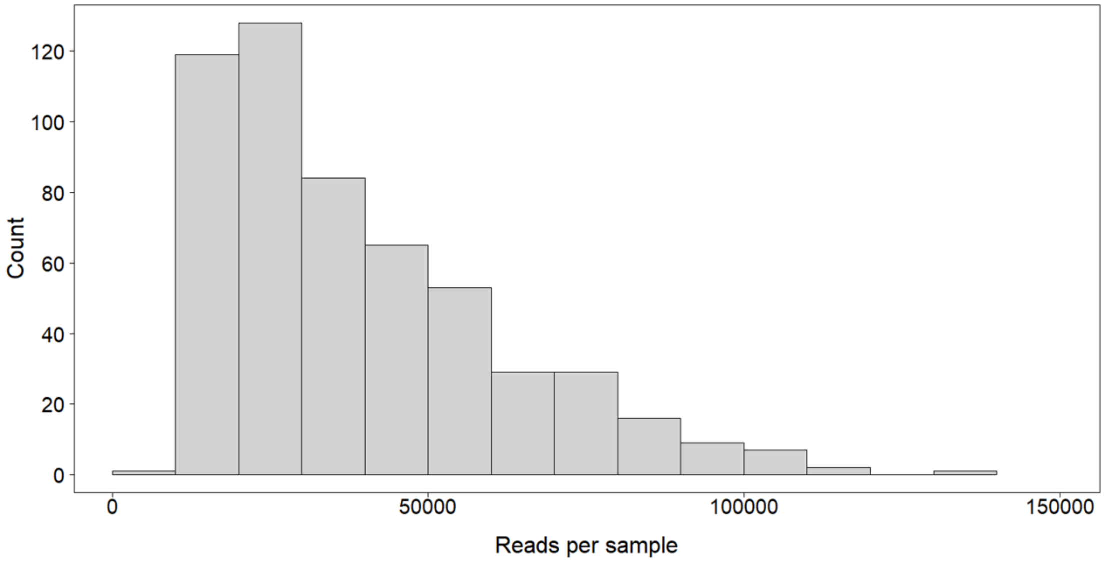

Figure S7. Number of reads per sample.

Diversity of gut bacterial communities and age

Reproductive status of the adult female

Onset of gestation was determined *a posteriori* by subtracting the average gestation length (i.e., 164 days, *Fürtbauer et al. 2010 IJP*) from the date of parturition. When exact date of parturition was unknown (24 out of 49 cases), the peak of the birth season (15th of May, *Fürtbauer et al. 2010 IJP*; unpublished data) was chosen and the conception set at the beginning of December of the previous year. Whether the female was gestating or not at the time of fecal sample collection was coded as a binary variable with the reference set to non-gestating.

Relationship between age, personal gut bacterial signature, and stability over time

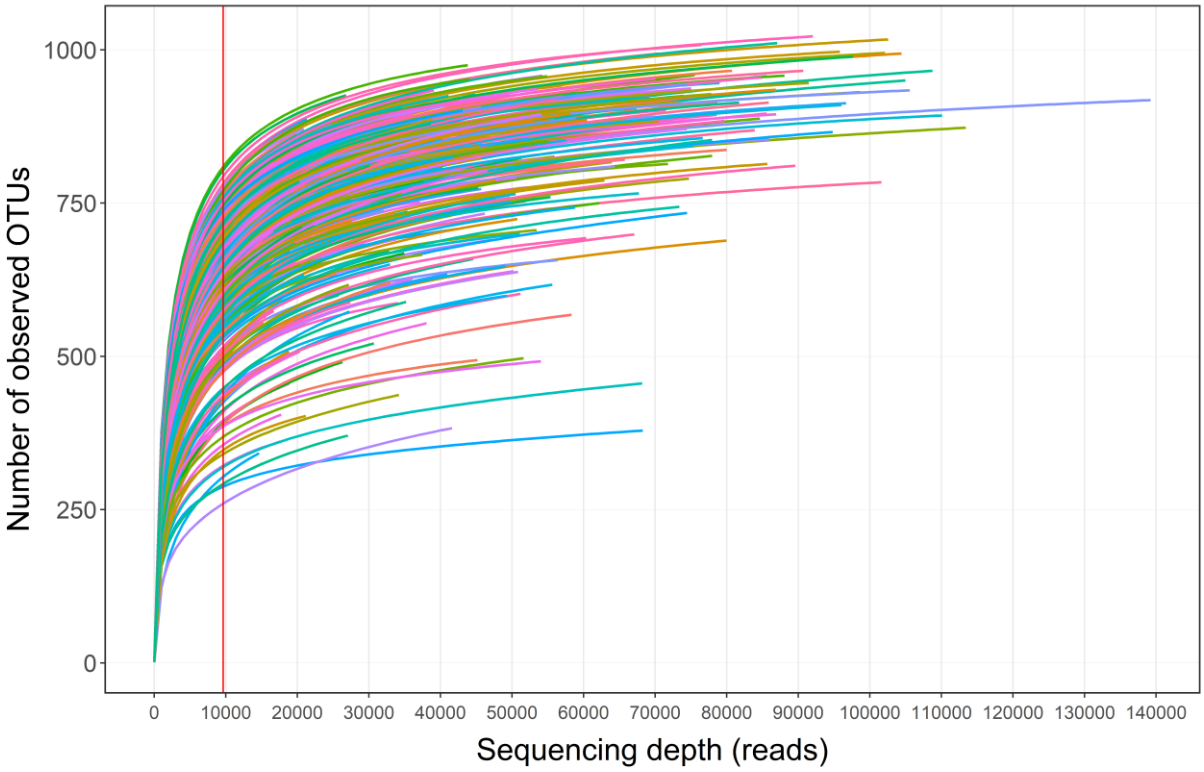

Figure S8. Rarefaction curves. Analyses were run on the dataset at 9653 reads (red vertical line).

References for R environment and packages used for analyses and plotting

1. Andersen, K. S., Kirkegaard, R. H., Karst, S. M. & Albertsen, M. *ampvis2: an R package to analyse and visualise 16S rRNA amplicon data*. 299537 https://www.biorxiv.org/content/10.1101/299537v1 (2018) doi:10.1101/299537.
2. Attali, D. & Baker, C. *Extra: Add Marginal Histograms to 'ggplot2', and More 'ggplot2' Enhancements. R package version 0.9.* (2019).
3. Auguie, B. *gridExtra: Miscellaneous Functions for 'Grid' Graphics. R package version 2.3.* (2017).
4. Bates, D., Mächler, M., Bolker, B. & Walker, S. Fitting Linear Mixed-Effects Models Using lme4. *J. Stat. Softw.* **67**, 1–48 (2015).
5. Brooks, M., E. *et al.* glmmTMB Balances Speed and Flexibility Among Packages for Zero-inflated Generalized Linear Mixed Modeling. *R J.* **9**, 378 (2017).
6. Bürkner, P.-C. brms: An R Package for Bayesian Multilevel Models Using Stan. *J. Stat. Softw.* **80**, 1–28 (2017).
7. Chen, J. *GUniFrac: Generalized UniFrac Distances, Distance-Based Multivariate Methods and Feature-Based Univariate Methods for Microbiome Data Analysis. R package version 1.3.* (2021).

8. Firke, S. *janitor: Simple Tools for Examining and Cleaning Dirty Data. R package version 2.1.0.* (2021).
9. Fox, J., Friendly, M. & Weisberg, S. Hypothesis tests for multivariate linear models using the car package. *R J.* **5**, 39–52 (2013).
10. Fox, J. & Weisberg, S. *An R Companion to Applied Regression*. (SAGE Publications, 2018).
11. Grolemund, G. & Wickham, H. Dates and Times Made Easy with lubridate. *J. Stat. Softw.* **40**, 1–25 (2011).
12. Kassambara, A. *ggpubr: 'ggplot2' Based Publication Ready Plots. R package version 0.4.0.* (2020).
13. Kembel, S. W. *et al.* Picante: R tools for integrating phylogenies and ecology. *Bioinformatics* **26**, 1463–1464 (2010).
14. Kuznetsova, A., Brockhoff, P. B. & Christensen, R. H. B. lmerTest Package: Tests in Linear Mixed Effects Models. *J. Stat. Softw.* **82**, 1–26 (2017).
15. Oksanen, J. *et al. vegan: Community Ecology Package. R package version 2.5-7.* (2020).
16. Paradis, E. & Schliep, K. ape 5.0: an environment for modern phylogenetics and evolutionary analyses in R. *Bioinformatics* **35**, 526–528 (2019).
17. R Core Team. *R: A language and environment for statistical computing. R Foundation for Statistical Computing, Vienna, Austria.* (2020).
18. RStudio Team. *RStudio: Integrated Development for R. RStudio, PBC, Boston, MA*. (2020).
19. Schliep, K., Potts, A. J., Morrison, D. A. & Grimm, G. W. Intertwining phylogenetic trees and networks. *Methods Ecol. Evol.* **8**, 1212–1220 (2017).
20. Wickham, H. The Split-Apply-Combine Strategy for Data Analysis. *J. Stat. Softw.* **40**, 1–29 (2011).
21. Wickham, H. Reshaping Data with the reshape Package. *J. Stat. Softw.* **21**, 1–20 (2007).
22. Wickham, H. *et al.* Welcome to the Tidyverse. *J. Open Source Softw.* **4**, 1686 (2019).
23. Wilke, C. *cowplot: Streamlined Plot Theme and Plot Annotations for 'ggplot2'. R package version 1.1.1.* (2020).
24. Xiao, N. *ggsci: Scientific Journal and Sci-Fi Themed Color Palettes for 'ggplot2'. R package version 2.9.* (2018).
